# A novel 450-nm laser-mediated sinoporphyrin sodium-based photodynamic therapy induces autophagic cell death in gastric cancer through regulation of the ROS/PI3K/Akt/mTOR signaling pathway

**DOI:** 10.1186/s12916-022-02676-8

**Published:** 2022-12-08

**Authors:** Xing Li, Lijiang Gu, Yuhang Chen, Xiaobing Wang, Yibo Mei, Jinlai Zhou, Minghai Ma, Jianbin Ma, Yue Chong, Xinyang Wang, Peng Guo, Dalin He, Jin Zeng

**Affiliations:** 1 Department of Urology, The First Affiliated Hospital of Xi’an Jiaotong University, 277 Yan-ta West Road, Xi’an, 710061 Shaanxi China; 2 Key Laboratory of Medicinal Resources and Natural Pharmaceutical Chemistry, Ministry of Education, National Engineering Laboratory for Resource Developing of Endangered Chinese Crude Drugs in Northwest of China, College of Life Sciences, Shaanxi Normal University, Xi’an, 710062 Shaanxi China; 3 Key Laboratory for Tumor Precision Medicine of Shaanxi Province, Xi’an, 710061 Shaanxi China; 4 Oncology Research Lab, Key Laboratory of Environment and Genes Related to Diseases, Ministry of Education, Xi’an, 710061 Shaanxi China

**Keywords:** PDT, GC, ROS, Autophagy, Apoptosis

## Abstract

**Background:**

Photodynamic therapy (PDT) has become an ideal and promising therapeutic method for fighting cancer, but its common application in clinical practice is prevented by the limitations of expensive devices in light sources and phototoxicity in photosensitizers. The aim of this study was to explore the antitumor efficiency of the novel 450-nm blue laser (BL) combined with sinoporphyrin sodium (DVDMS)-mediated PDT against human gastric cancer (GC) in vitro and in vivo, focusing on autophagy pathway.

**Methods:**

Cell viability was detected by Cell Counting Kit-8 and colony formation assays in HGC27, MGC803, AGS, and GES-1 cells. Cell apoptosis was measured by flow cytometry and western blotting. The production of reactive oxygen species (ROS) was measured by fluorescence microscopy and flow cytometry. Autophagy was determined by transmission electron microscopy and western blotting. The antitumor effect of BL-PDT in vivo was detected by a subcutaneous tumor model in nude mice.

**Results:**

The novel 450-nm laser-mediated DVDMS-based PDT caused remarkable growth inhibition and apoptosis induction in GC cells in vitro by the production of excessive ROS. Autophagy flux was induced by BL-PDT in GC cells, as determined by LC3 conversion assay, LC3 turnover assay, and mRFP-GFP-LC3 puncta assay. Furthermore, autophagy induction was demonstrated to positively contribute to BL-PDT-induced apoptotic effects on GC cells. Mechanically, ROS/PI3K/Akt/mTOR pathway was identified to involve in the regulation of BL-PDT-induced autophagy as determined by transcriptomic analysis and functional studies. Consistently, xenograft studies confirmed the significant antitumor effect of BL-PDT and its favorable safety in vivo.

**Conclusions:**

The novel 450-nm laser-mediated DVDMS-based PDT may be a safe and effective approach against GC. Our results thus provide compelling evidence for the therapeutic application of BL-PDT in human GC.

**Supplementary Information:**

The online version contains supplementary material available at 10.1186/s12916-022-02676-8.

## Background

Gastric cancer (GC) is one of the most common and intractable malignant tumors worldwide; it has high morbidity and mortality and seriously affects the physical and mental health of human beings [1, 2]. According to statistics, there were over a million new cases of GC and seven hundred thousand deaths (equating to one in every twelve deaths) from the disease worldwide in 2018 [3]. However, these statistics are far higher in East Asian countries (especially Korea, Japan, and China) than in North America, Northern Europe, and African regions due to environmental and dietary differences [4, 5]. In recent years, with the progress of endoscopic technology and diagnostic methods, an increasing number of GC patients have been identified, but many sufferers are in the middle and late stages owing to the lack of early symptoms [1]. Surgical operation with curative intent is the primary therapeutic regimen for patients with early-stage GC, while palliative chemoradiotherapy and molecular targeted therapy are regarded as the main therapeutic options for advanced patients [6, 7]. Despite the constant innovations and improvements in the treatment of GC, the prognosis of patients is still not optimistic, especially in advanced stages [8]. Thus, to enhance the quality of life and survival time of patients with GC, there is an urgent need to explore more effective and safe innovative treatments for GC.

Photodynamic therapy (PDT) is an emerging and promising noninvasive treatment strategy for malignancy and other diseases, including cancer of the stomach [9]. As early as 1900, the German scientist Raab found that combining a harmless dye (acridine) with visible light could kill paramecia, which was the first report of PDT and opened new avenues for the exploration of anticancer therapies [10]. Compared with traditional antitumor therapies, PDT shows strong selective toxicity for cancer cells and satisfactory safety for normal tissues, as well as less resistance and no long-term side effects [11]. PDT takes advantage of the cytotoxicity of reactive oxygen species (ROS) that are generated by a specific wavelength light irradiating some kind of photosensitizers in the presence of sufficient oxygen [12]. Of note, these components are not cytotoxic when present alone, and only when combined will they lead to tumor cell death [13]. Although clinical studies of PDT in the treatment of GC have been reported before, it cannot be widely promoted in clinical practice due to the limitations of expensive devices in light sources and phototoxicity in photosensitizers [14–16]. Therefore, it is particularly important to explore novel light sources and photosensitizers of PDT.

In terms of photosensitizers, good chemical stability, high water solubility and photodynamic effect, low dark toxicity, and rapid clearance in patients should be the central characteristics of an ideal photosensitizer [12, 17]. As a novel sodium salt of the ether-linked porphyrin dimer, sinoporphyrin sodium (DVDMS) was isolated and purified from Photofrin II and showed great potential in PDT [18, 19]. DVDMS has stronger photodynamic effect and lower skin phototoxicity than traditional photosensitizers such as Photofrin and Hiporfin in several xenograft tumors, which has also been proven in our previous studies [20–23]. In addition, DVDMS has better water solubility and chemical stability and has been approved by the Chinese State Food and Drug Administration for performing phase II clinical trials in some advanced tumors.

In terms of light sources, the commonly used light source of PDT in clinical practice and research is a red laser (RL) with a 630-nm wavelength, which shows strong tissue penetration but is prone to energy attenuation, especially in a liquid environment [24]. Based on the long-term systematic and in-depth study of laser characteristics with different wavelengths, our team successfully developed the world’s first 450-nm laser surgery system, which can not only be used for vaporization and cutting in surgery, but can also serve as a new type of light source for PDT, and has been approved by the China National Medical Products Administration (20213010922). Our previous studies have shown that the 450-nm laser has the advantages of high-efficiency tissue vaporization, low laser tissue penetration, and good tissue solidification in different tissue-cutting processes, which could be a novel alternative to surgery for superficial diseases [25–27]. Based on the safety and efficacy of this wavelength laser, we intend to explore the effect of a low-dose 450-nm laser in PDT of superficial intracavitary tumors. From our previous investigation of the absorption spectra of porphyrin compounds, we found that the porphyrin photosensitizer has a good absorption effect at the wavelength of 450 nm [18]. In this study, using the novel light source and effective photosensitizer, we first explored the potential antitumor effect of the 450-nm laser-mediated DVDMS-based PDT for human gastric cancer and investigated the underlying molecular mechanism focusing on autophagic and apoptotic signaling pathway.

## Methods

### Sensitizers

**Fig. 1:**
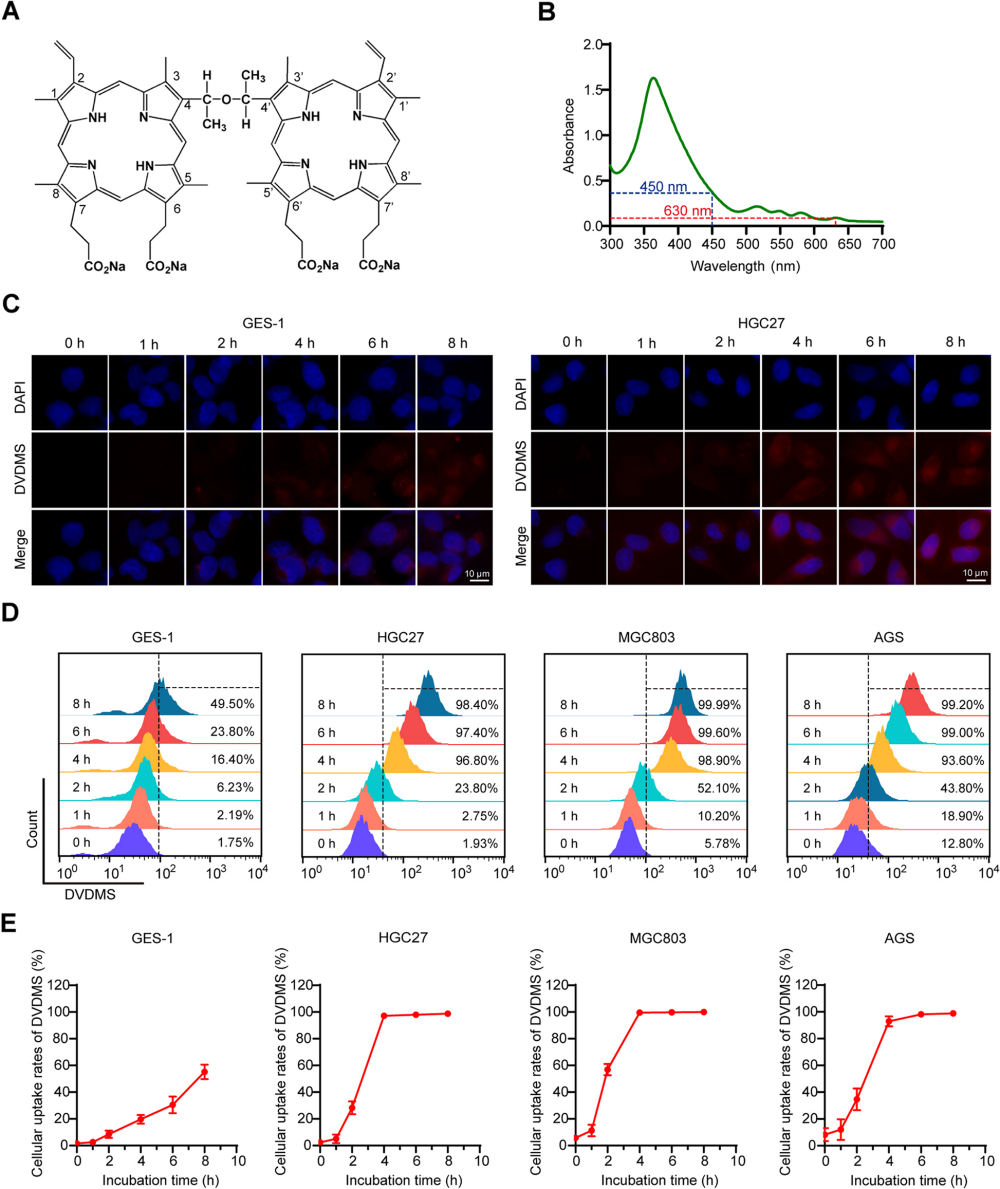
The chemical structure, absorption spectrum, and cellular uptake of DVDMS. **A** The chemical structure of DVDMS. **B** The absorption spectrum of DVDMS. **C** Representative fluorescence images of the intracellular uptake of DVDMS after different incubation time points. Scale bar = 10 μm. **D**, **E** Flow cytometry analysis of the intracellular uptake of DVDMS for various times (*n* = 3, mean ± SD)

Sinoporphyrin sodium (DVDMS, molecular formula: C_68_H_66_N_8_O_9_Na_4_, molecular weight: 1230.265 Da, purity: 98.5%) was produced by Jiangxi Qinglong High Technology Co., Ltd. (Yichun, China), dissolved in 0.9% sodium chloride to form a solution with a final concentration of 1.25 mg/mL, sterilized by a 0.22-μm filter, and stored in the dark at −20 °C. The chemical structure and absorption spectrum of DVDMS are presented in Fig. 1A, B.

### Cell culture

The human GC cell lines HGC27 and AGS were purchased from the Cell Bank of Type Culture Collection of the Chinese Academy of Sciences (Shanghai, China), and the human GC cell line MGC803 and human gastric epithelium cell line GES-1 were obtained from the Key Laboratory for Tumor Precision Medicine of Shaanxi Province (Xi’an, China). HGC27 and MGC803 cells were cultured in the RPMI 1640 medium (Sigma-Aldrich, WI, USA), AGS cells were maintained in Ham’s/F-12 medium (Procell, Wuhan, China), and GES-1 cells were grown in Eagle’s minimum essential medium (DMEM, Sigma-Aldrich, WI, USA), containing 10% fetal bovine serum (Biological Industries, Israel) and 1% penicillin/streptomycin (New Cell & Molecular Biotech, Suzhou, China). All cells were incubated in a water-saturated atmosphere of 5% CO_2_ at 37 °C and were collected at the peak of the logarithmic growth phase for experiments.

### Reagents and irradiation

The Cell Counting Kit-8 (CCK-8) assay kit was purchased from TargetMol (MA, USA), 5-ethynyl-2′-deoxyuridine (EdU) assay kit was from Beyotime (Shanghai, China), N-acetylcysteine (NAC) was from Selleck (Shanghai, China), and 2′,7′-dichlorodihydrofluorescein diacetate (DCFH-DA) was from KeyGen BioTECH (Nanjing, China). The Annexin V-FITC/PI Apoptosis detection kit was purchased from BD Bioscience (Franklin Lakes, NJ, USA), chloroquine (CQ) phosphate salt was from Sigma-Aldrich (Germany), and 2-(4-Amidinophenyl)-6-indolecarbamidine dihydrochloride (DAPI) was from Solarbio (Beijing, China). We obtained 3-methyladenine (3-MA), LY294002, and 740 Y-P from MedChemExpress (Monmouth Junction, NJ, USA). All the other reagents were analytical grade commercial products.

Semiconductor lasers (excitation wavelength: 450 nm and 630 nm, respectively; manufacturer: Blueray Medical Ltd., Xi’an, China) were carried out as the source for the evocation of the photodynamic effect.

### Detection of intracellular DVDMS uptake

All cell lines (HGC27, MGC803, AGS, and GES-1) were planted in 24-well plates (1 × 10^5^ cells/well) and 6-well plates (3 × 10^5^ cells/well), respectively, and incubated with DVDMS (1 μM) in the dark at 37 °C for indicated time periods. For measurement of the intracellular uptake of DVDMS, cells were collected at different incubation time points (0, 1, 2, 4, 6, 8 h) and detected with flow cytometry (FACSCalibur, BD, USA) and fluorescence microscopy (Olympus, Japan). The mean fluorescence intensity (MFI) of DVDMS in cells was recorded under uniform detection conditions.

### Cell viability assay

Cell viability was estimated using CCK-8, colony formation, and EdU assays. The GC cell lines HGC27, MGC803, AGS, and gastric epithelium cell line GES-1 were cultured in 96-well plates (5 × 10^3^ cells/well) and incubated with DVDMS at various concentrations (0, 0.05, 0.1, 0.2, and 0.4 μM) at 37 °C in darkness for 4 h and then exposed to an energy density of 10 mW/cm^2^ in a BL (wavelength 450 nm) for 600 s to achieve a final laser dose of 6 J/cm^2^. Meanwhile, DVDMS alone with the same concentration gradient was incubated separately to detect the cytotoxicity. After PDT for 24 h, CCK-8 was mixed with each well to detect cell viability and GraphPad Prism 8.0 software was performed to calculate the IC_50_. Notably, the IC_50_ of each cell line was selected as the appropriate incubation concentration of DVDMS in subsequent experiments. And then, the PDT effects of BL (450 nm) and RL (630 nm), or DVDMS and 5-aminolevulinic acid (5-ALA) on cells were compared under the same drug dose (IC_50_) and irradiation power (6 J/cm^2^), and morphological changes in the cells were observed under a microscope. In addition, the light dose correction (LDC) of BL and RL should be considered according to the method proposed by Schaberle [28].

A colony formation assay was carried out to assess the long-term proliferative potential of all GC cell lines after PDT. Meanwhile, the maximum IC_50_ of GC cell lines was used as the incubation concentration of DVDMS to observe the PDT effect on the proliferation of normal gastric mucosal cells. The treated cells were seeded in 6-well plates (600 cells/well) and cultured for 10 days. During this period, the culture medium was replaced with a fresh medium every 3 days. After visible colonies formed, cells were fixed in 4% paraformaldehyde (PFA) for 10 min and incubated with 0.1% crystal violet solution for 20 min. The number of stained colonies containing more than 50 cells was observed and manually counted under a microscope.

For the EdU assay, cells were seeded in 24-well plates at a density of 1 × 10^5^ cells/well. After PDT, the cells were incubated with EdU working solution for 2 h, fixed with 4% PFA, and permeated with 0.3% Triton X-100 PBS. And then, a series of reaction solutions were added to the well and the nuclei were finally stained with Hoechst. After that, the results of cell proliferation were visualized by a fluorescence microscope.

### Measurement of intracellular ROS production

Briefly, cells were incubated with serum-free medium containing 10 μM DCFH-DA in the dark for 30 min prior to PDT. After treatment, the fluorescence intensity was observed immediately with a fluorescence microscope and then detected by flow cytometry. In addition, an electron spin resonance (ESR) spectrometer (A300-9.5/12, Bruker, Germany) was used to detect the different types of ROS, and 2,2,6,6-tetramethyl-4-piperidone (TEMP) and 5,5-dimethyl-1-pyrroline-N-oxide (DMPO) were selected as capture agents to identify singlet oxygen (^1^O_2_) and hydroxyl radicals (·OH), respectively.

For inhibitory experiments, cells were preincubated with 5 mM NAC (a ROS scavenger) for 1 h and washed with PBS three times after 1 h of PDT. Then, the fluorescence intensity and cell viability were assessed using the same method as above.

### RNA sequencing

According to the instructions of Novogene Co., Ltd. Tianjin Sequencing Center, the total RNA extracts of HGC27 and MGC803 cells treated with BL-PDT were separated with TRIzol, isolated by chloroform, and extracted using ethanol, respectively. The cDNA library construction, library purification, and transcriptome sequencing were then carried out for deep data analysis. Of note, three duplicate samples from each group were used for RNA-sequencing analysis.

### Apoptosis assay

The apoptotic ratios of HGC27, MGC803, and AGS cells treated with BL-PDT were detected via flow cytometry. The cells were respectively implanted into 6-well plates at a density of 3 × 10^5^ cells/well. After BL-PDT for 24 h, the cells were washed twice with precooled PBS, collected in 1.5-mL tubes, and centrifuged at 500 g (4 °C) for 5 min. Subsequently, the cells were resuspended in 100 μL of a binding buffer and mixed with 4 μL of Annexin V-FITC and 4 μL of propidium iodide (PI) for 15 min. After that, 400 μL of binding buffer was added for dilution and flow cytometry was performed to measure the proportions of apoptotic cells.

### Transfection and evaluation of fluorescent dots

According to the instructions of Hanbio Co., Ltd. (Shanghai, China), all GC cells were seeded in 6-well plates at a density of 20 × 10^5^ cells/well overnight. The appropriate concentrations of mRFP-GFP-LC3 adenovirus and polyethyleneimine were mixed and incubated for 20 min at room temperature and then slowly added to the serum-free medium, which was used to replace the original cell culture medium. After 24 h of transfection, the fresh serum medium was replaced. After 4 h of stabilization, the cells were digested by trypsin and planted in 24-well plates containing climbing sheets. After the growth of cell adhesion, PDT was performed. Then, the cells were incubated in the dark at 37 °C for 24 h, observed under a confocal fluorescence microscope, and analyzed by ImageJ software. In merged images, yellow fluorescent spots in transfected cells demonstrated autophagosomes, red spots indicated autophagic lysosomes, and the enhancement of both yellow and red fluorescent dots indicated increased autophagic flux.

### Transmission electron microscopy (TEM) analysis

After treatment with BL-PDT for 12 h, the cells were harvested and fixed with ice-cold 2.5% glutaraldehyde for 2 h at 4 °C. Then, the fixed cells were post-treated with 1% buffered osmium tetroxide for 1 h at 4 °C and dehydrated by subsequently passing through a graded series of ethanol. After fixation and embedding, the cell samples were sectioned into thin pieces, stained with 3% uranyl acetate/lead citrate, and finally visualized with an H-7650 TEM (Hitachi, Japan).

### Western blot assay

The expression levels of involved proteins were measured by western blotting analysis and analyzed by ImageJ software. Briefly, the cell samples at 24 h after BL-PDT were collected and lysed in 1 × radioimmunoprecipitation assay buffer with 1% phenylmethylsulfonyl fluoride for 20 min on ice. After quantification by a bicinchoninic acid assay kit (Beyotime, Shanghai, China), lysates were boiled for 10 min at 100 °C, and aliquots of 20 μg of protein were separated by 10% or 12% sodium dodecyl sulfate-polyacrylamide gel electrophoresis and transferred onto polyvinylidene difluoride membranes that were then blocked with 5% skim milk for 1 h at room temperature and incubated with primary antibodies on a shaker (4 °C, overnight). After 3 times of washing with TBST and 2 h of incubation with the corresponding secondary antibody at room temperature, the immunoblots on the membranes were observed using an enhanced chemiluminescence detection kit (Millipore, Burlington, MA, USA). The primary antibodies included LC3 and PARP antibodies (all from Cell Signaling Technology, USA); p62, Beclin-1, Bax, Bcl-2, Caspase 3, and Caspase 9 antibodies (all from Abcam, UK); and PI3K, p-PI3K, Akt, p-Akt, mTOR, and p-mTOR antibodies (all from Abmart, China). Mouse anti-human β-Actin antibody and goat anti-mouse or anti-rabbit secondary antibody were purchased from Abcam.

### Animal experiment

BALB/c mice (age 4–5 weeks, male, 18–20 g body weight) were purchased from the Shanghai Experimental Animal Center of the Chinese Academic of Sciences (Shanghai, China) and housed in an air-conditioned animal center at 22 °C ± 2 °C with free access to food and water. All in vivo experimental procedures were approved by the ethics committee of Xi’an Jiao Tong University (Xi’an, China). After 1 week of adaptation, 6 × 10^6^ MGC803 cells were subcutaneously injected into the right dorsal region of BALB/c mice. The body weight and tumor size of each animal were measured every other day, and the total volume was calculated as *V* = (length × width^2^)/2. When tumors reached an average volume of 100 mm^3^ (approximately 10–12 days post-injection), the mice were randomly divided into four groups: (1) control (0.9% saline, 0.1 mL), (2) 450-nm laser (60 J/cm^2^), (3) DVDMS (2 mg/kg, 0.1 mL), and (4) PDT (DVDMS + 450-nm laser). DVDMS was injected into the caudal vein, and irradiation was performed 24 h after injection [18]. Fourteen days after treatment, all the mice were sacrificed, and the entire tumors were collected and weighed.

In addition, the effects of 450-nm and 630-nm laser-mediated DVDMS-PDT on GC growth were also compared in vivo. The mice were randomly divided into three groups: (1) DVDMS (2 mg/kg, 0.1 mL), (2) RL-PDT (DVDMS + 630-nm laser (60 J/cm^2^)), and (3) PDT (DVDMS + 450-nm laser (60 J/cm^2^)), and the remaining steps were the same as above.

### Hematoxylin and eosin (H&E) staining and immunohistochemistry (IHC) analysis

After being fixed in 4% PFA for at least 24 h, the tumor tissue and major organs of each animal were embedded in paraffin, sliced into thick sections, and stained with H&E. Histopathological changes were observed with an inverted fluorescence microscope. For IHC examination, sliced tumor tissues were incubated with primary antibodies (LC3-II, p-Akt, and Ki-67) for 20 h at 4 °C and secondary antibody for 1 h. After visualization with diaminobenzidine solution conjugated with hematoxylin, the images were observed with a microscope and analyzed by ImageJ software.

### Statistical analysis

All quantitative results are expressed as the mean ± standard deviation (SD). Statistical analysis was carried out with GraphPad Prism 8.0 software by the two-tailed unpaired Student’s *t* test and log-rank test. Statistical significance is expressed as *p < 0.05, **p < 0.01, and ***p < 0.001.

## Results

### Intracellular uptake of DVDMS

The intracellular uptake of DVDMS was assessed by detecting the MFI using fluorescence microscopy and flow cytometry. The red fluorescence of DVDMS was obviously detected in GC cells after incubation for 4 h (Fig. 1C and Additional file 1: Fig. S1A-B). Similarly, the results of flow cytometry showed that the accumulation of DVDMS in GC cells (HGC27, MGC803, and AGS) was more than 90% after 4 h of incubation (Fig. 1D, E). Conversely, the accumulation in normal gastric epithelial cell (GES-1) increased slowly, when compared with cancer cells (Fig. 1D, E). Thus, 4-h incubation of DVDMS was chosen for further experiments.

### Cytotoxic and anti-proliferative effects of PDT on GC cells

**Fig. 2:**
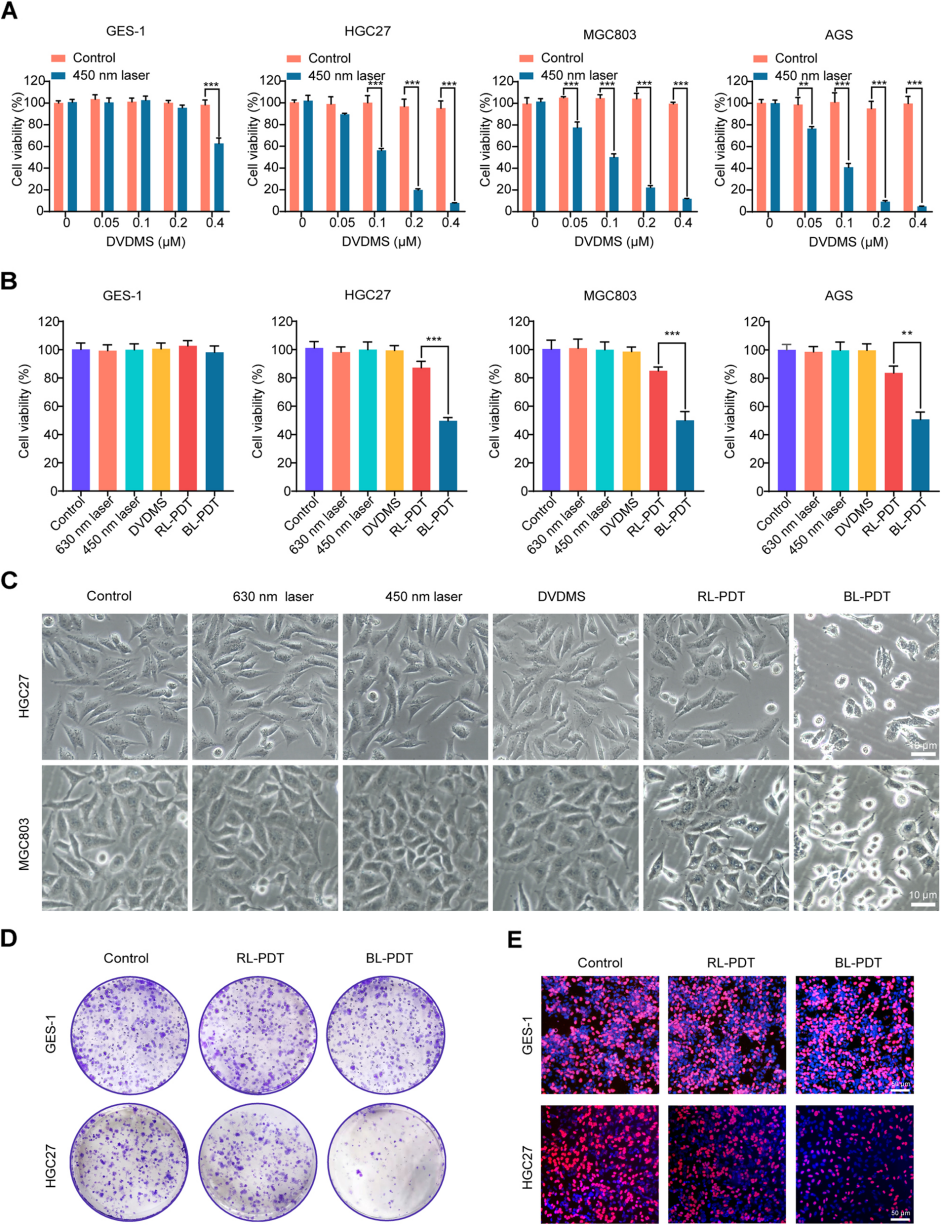
Anti-proliferative effects of PDT on GC cells. **A** Photodynamic effect and cytotoxicity of DVDMS on cells for 24 h (*n* = 3, mean ± SD). **B** Comparison of cytotoxic effect after different treatments for 24 h (*n* = 3, mean ± SD). **C** Representative morphology images of GC cells after different treatments for 24 h. Scale bar = 10 μm. **D** Colony formation test after treating with BL-PDT and RL-PDT. **E** EdU assay after treating with BL-PDT and RL-PDT. Scale bar = 50 μm. *p < 0.05, **p < 0.01, and ***p < 0.001

Next, we aimed to determine the PDT effects of the novel 450-nm laser (BL) combined DVDMS on GC. As clearly seen in Fig. 2A, PDT significantly inhibited GC cell proliferation in a DVDMS dose-dependent manner, and the proliferation of GES-1 cells was not significantly inhibited by PDT. And DVDMS at the same concentration alone without irradiation did not show obvious cytotoxicity in any cell line. According to the cell viability assay data, the IC_50_ of each cell line was calculated and used in subsequent experiments (HGC27: 0.13 μM; MGC803: 0.12 μM; AGS: 0.084 μM). Of note, a combination of BL and DVDMS showed better PDT effects than traditional 630 nm (RL) and 5-ALA under the same conditions (Fig. 2B and Additional file 1: Fig. S1C). Morphologically, more obvious changes, such as condensed chromatin and nuclear fragmentation, were observed in the BL-PDT group than in the RL-PDT group, while no visible changes were found in the control, laser, or DVDMS alone groups (Fig. 2C). The strong anti-proliferative effects of BL-PDT on GC cells were further confirmed by colony formation test and EdU assay (Fig. 2D, E and Additional file 1: Fig. S1D-F). Interestingly, the proliferation ability of normal gastric mucosal was not affected by any type of PDT (Fig. 2D, E and Additional file 1: Fig. S1D-F).

Of note, the real effect in PDT is recommended to be evaluated by the actual light dose absorbed by the photosensitizer, which can be calibrated by the relative parameter LDC. According to the method proposed by Schaberle [28], the LDC values of BL and RL are calculated as 0.48 (BL) and 0.11 (RL), respectively. Hence, to achieve the same PDT effect as BL, the output energy of RL needs to be increased by more than 4 times.

### PDT induces ROS production in GC cells

**Fig. 3:**
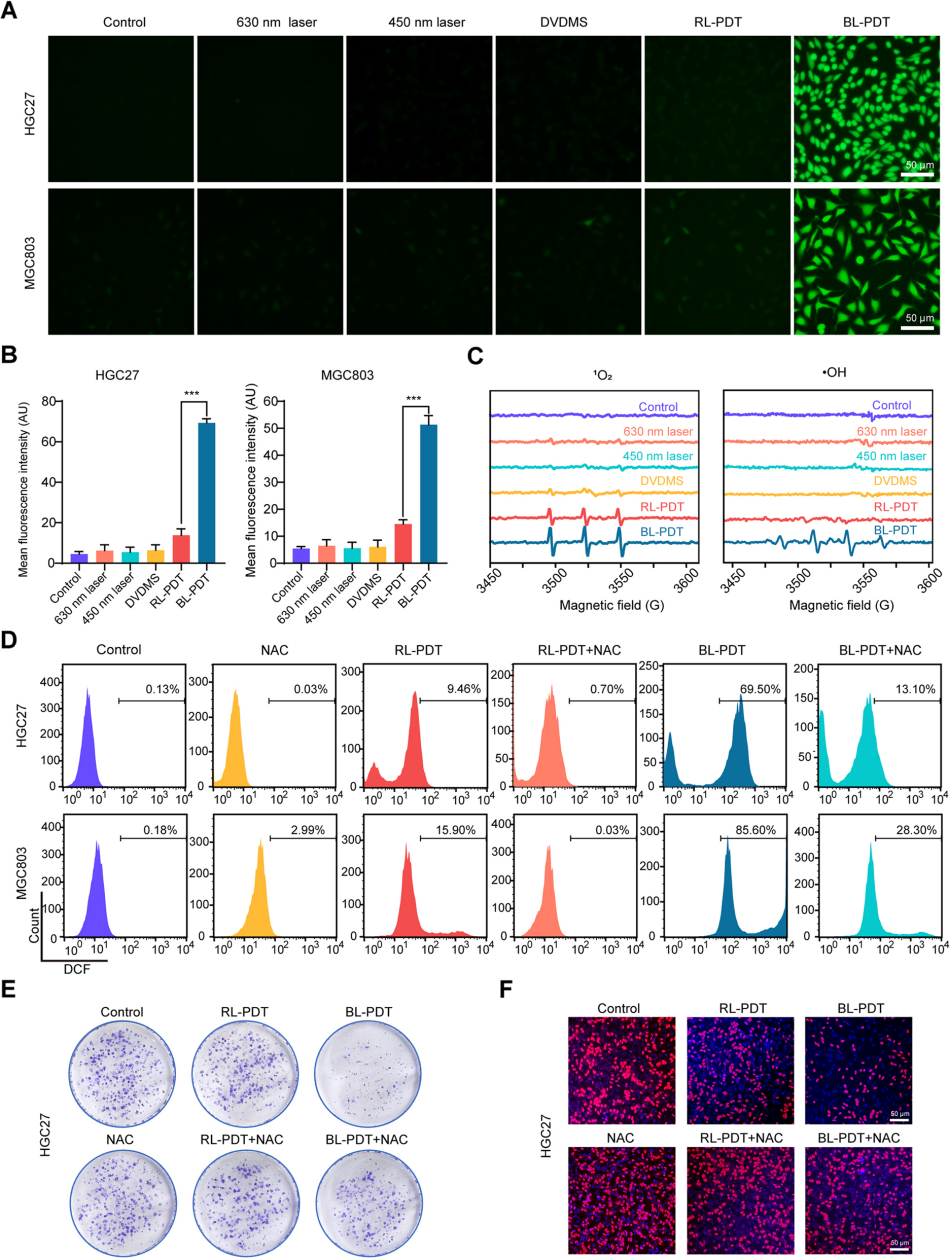
Effects of PDT on ROS production in GC cells. **A**, **B** Representative fluorescence images and quantitative analysis of intracellular ROS detection. Scale bar = 50 μm (*n* = 3, mean ± SD). **C** ESR spectra of TEMP/^1^O_2_ and DMPO/·OH after different treatments. **D** Flow cytometry analysis of the ROS amount after adding NAC (incubation 2 h). **E** Colony formation test of HGC27 cells after adding NAC (incubation 2 h). **F** EdU assay of HGC27 cells after adding NAC (incubation 2 h). Scale bar = 50 μm. *p < 0.05, **p < 0.01, and ***p < 0.001

It is well known that the mechanistic basis of cell damage induced by PDT is closely associated with the generation of intracellular ROS. Consistent with previous studies, we found that BL-PDT significantly increased the fluorescence intensity of the intracellular ROS probe compared to the other treatments under the fluorescence microscope (Fig. 3A, B). Moreover, the ESR results indicated that more ^1^O_2_ and ·OH were produced in the BL-PDT group (Fig. 3C). Furthermore, the results of flow cytometry and fluorescence microscopy showed that the amount of ROS in the BL-PDT group decreased significantly after adding the ROS scavenger NAC (Fig. 3D and Additional file 1: Fig. S2A-B). Functionally, inhibitory effects of BL-PDT on the cell survival rate and proliferation rate were greatly rescued by NAC, suggesting the vital role of ROS in mediating cell growth inhibition (Fig. 3E, F and Additional file 1: Fig. S2C-E).

### Differentially expressed genes and enrichment analysis in GC cells after 450-nm laser/DVDMS-mediated PDT

**Fig. 4:**
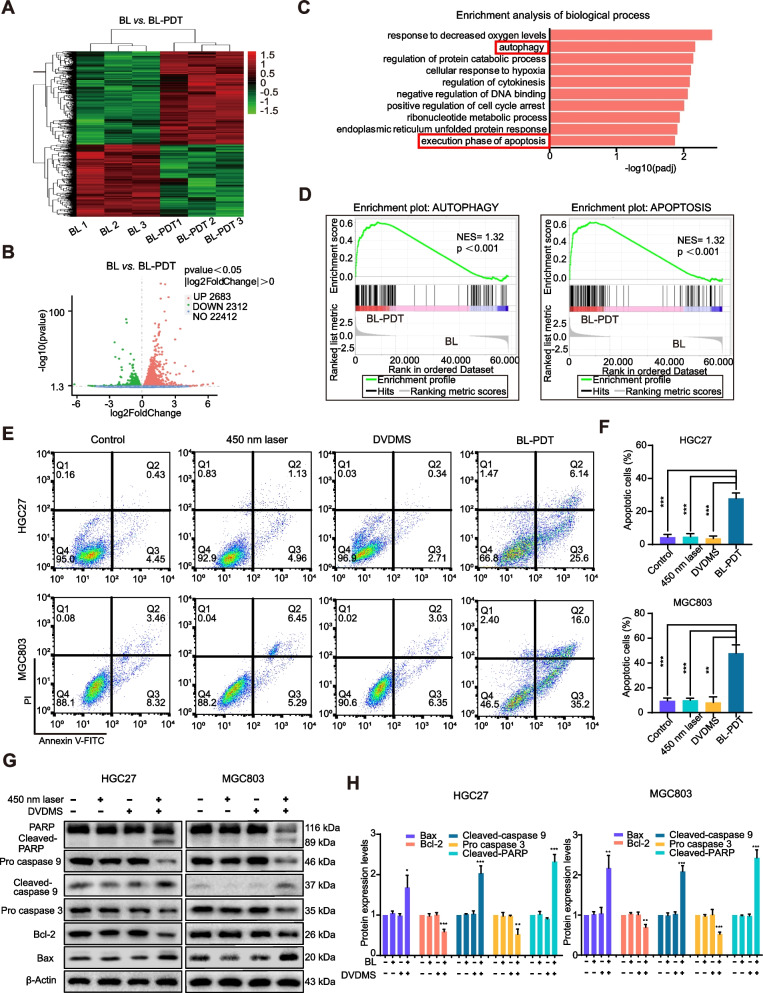
Apoptotic effects of 450-nm laser/DVDMS-mediated PDT on GC cells. **A**, **B** Heatmap and volcano plot of the DEGs in HGC27 cells between BL-PDT group vs. BL group. **C**, **D** GO and KEGG enrichment analysis of the DEGs in HGC27 cells. **E**, **F** Flow cytometry analysis of the apoptosis in GC cells after the treatment of 24 h (*n* = 3, mean ± SD). **G** Western blotting analysis of the expression levels of apoptosis-related proteins after the treatment of 24 h. **H** Quantitative analysis of **G** (*n* = 3, mean ± SD). *p < 0.05, **p < 0.01, and ***p < 0.001

To explore the molecular changes in GC cells induced by BL-PDT, RNA-seq was performed. The results showed that significant differences in gene expression occurred after treatment (Fig. 4A and Additional file 1: Fig. S3A), in which 4995 differentially expressed genes (DEGs) including 2683 upregulated genes and 2312 downregulated genes were identified in HGC27 cells, and 4417 DEGs including 2183 upregulated genes and 2234 downregulated genes in MGC803 cells as shown in the volcano plot (Fig. 4B and Additional file 1: Fig. S3B). Enrichment analysis by GO indicated that most of the DEGs were enriched in cell autophagy and apoptosis processes (Fig. 4C and Additional file 1: Fig. S3C), and similar results were also observed in the gene set enrichment analysis (GSEA) of KEGG (Fig. 4D).

### 450-nm laser/DVDMS-mediated PDT induces apoptosis in GC cells

To explore whether BL-PDT-mediated inhibition of GC cell growth is related to apoptosis, we performed Annexin V-FITC/propidium iodide (PI) double staining combined with flow cytometry to detect and analyze the apoptotic rate of cells. The results showed that the apoptotic cells in the BL-PDT group were significantly increased compared with the other groups (Fig. 4E, F and Additional file 1: Fig. S3D). Consistently, the western blotting analysis also demonstrated that BL-PDT downregulated the expression of Bcl-2, pro-caspase 3, pro-caspase 9, and PARP and upregulated the expression of Bax, cleaved-caspase 9, and cleaved-PARP in GC cells (Fig. 4G, H and Additional file 1: Fig. S3E-F).

### 450-nm laser/DVDMS-mediated PDT activates autophagy in GC cells

**Fig. 5:**
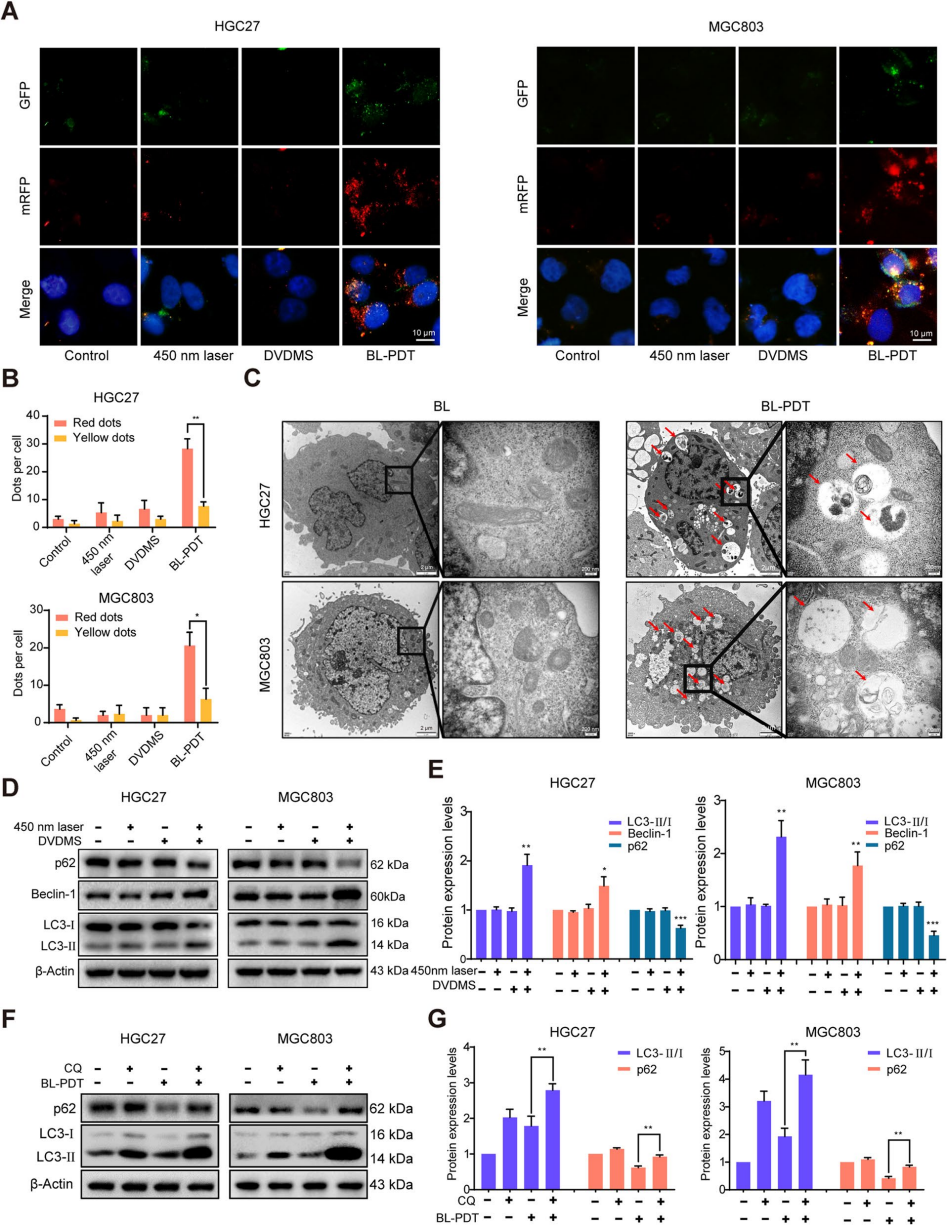
450-nm laser/DVDMS-mediated PDT induced autophagy flux in GC cells. **A** Representative fluorescence images of autophagy dots in GC cells after the treatment of 24 h. Scale bar = 10 μm. **B** Quantitative analysis of **A** (*n* = 3, mean ± SD). **C** Typical TEM images of the autophagic vacuoles (indicated by the red arrow) after PDT for 24 h. **D** Western blotting analysis of the expression levels of autophagy-related proteins after the treatment of 24 h. **E** Quantitative analysis of **D** (*n* = 3, mean ± SD). **F** Western blotting analysis of the effect of inhibitor CQ for 4 h on autophagy. **G** Quantitative analysis of **F** (*n* = 3, mean ± SD). *p < 0.05, **p < 0.01, and ***p < 0.001

Then, we investigated whether BL-PDT could initiate autophagy in the three GC cell lines. First, to assess the autophagic flux of GC cells upon BL-PDT, cells were transfected with an adenovirus encoding mRFP-GFP-LC3. Compared to the other groups, the BL-PDT group had a significantly increased number of red spots inside these three GC cell lines after 24 h of treatment, suggesting a complete autophagic flux induced by BL-PDT (Fig. 5A, B and Additional file 1: Fig. S4A). Then, the intracellular ultrastructure of the GC cells treated with BL-PDT was detected by TEM, which is a classic method for determining autophagy. And the typical autophagic double membrane-containing vacuoles were observed in the cytoplasm of GC cells after BL-PDT (Fig. 5C and Additional file 1: Fig. S4B). In the control group (450-nm laser alone), the morphology of the cytoplasm and organelles was normal, and no obvious autophagic vacuoles were observed.

Next, we explored the effect of BL-PDT on the expression of key autophagy-related molecules in GC cells by western blotting. The results indicated that BL-PDT significantly enhanced the accumulation of LC3-II and Beclin-1 and reduced the expression of p62 in GC cells (Fig. 5D, E and Additional file 1: Fig. S4C). Moreover, a strong accumulation of LC3-II was observed after adding the lysosomal inhibitor CQ, indicating the activation of autophagy (Fig. 5F, G, and Additional file 1: Fig. S4D).

### Autophagy positively contributes to 450-nm laser/DVDMS-mediated PDT-induced apoptosis in GC cells

**Fig. 6:**
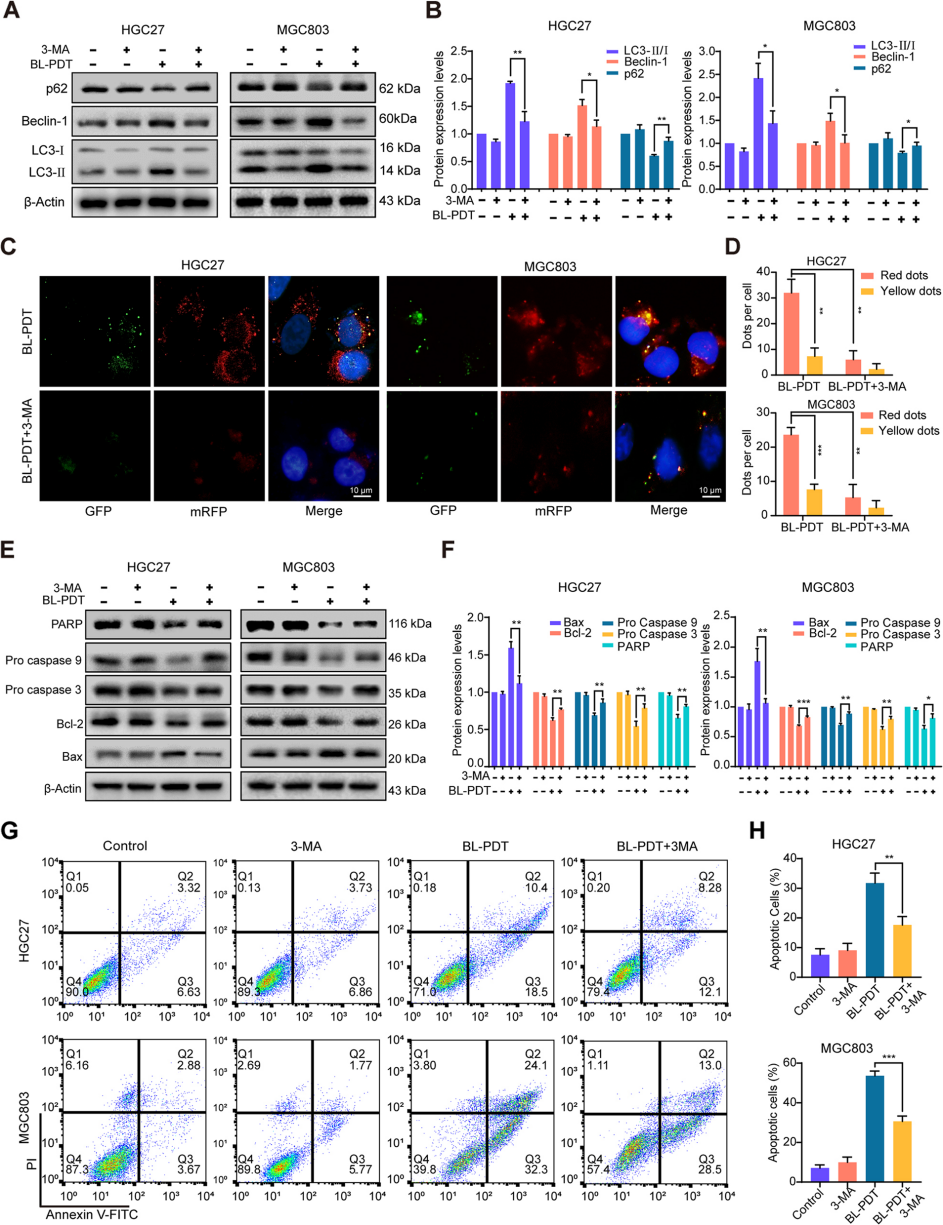
450-nm laser/DVDMS-mediated PDT induced autophagic cell death in GC cells. **A** Western blotting analysis of the effect of inhibitor 3-MA for 12 h on autophagy. **B** Quantitative analysis of **A** (*n* = 3, mean ± SD). **C** Representative fluorescence images of autophagy dots after adding inhibitor 3-MA. Scale bar = 10 μm. **D** Quantitative analysis of **C** (*n* = 3, mean ± SD). **E** Western blotting analysis of the effect of autophagy on BL-PDT inducing apoptosis after adding inhibitor 3-MA. **F** Quantitative analysis of **E** (*n* = 3, mean ± SD). **G**, **H** Flow cytometry analysis of the apoptosis after adding inhibitor 3-MA (*n* = 3, mean ± SD). *p < 0.05, **p < 0.01, and ***p < 0.001

To further illustrate the relationship between autophagy and apoptosis induced by BL-PDT, the inhibitor 3-MA of autophagy initiation was applied. The results showed that BL-PDT-induced downregulation of p62 and upregulation of Beclin-1 and LC-3 II were markedly reversed (Fig. 6A, B and Additional file 1: Fig. S4E). Consistently, the number of red fluorescent spots inside GC cells was decreased after adding 3-MA (Fig. 6C, D and Additional file 1: Fig. S4F). In addition, 3-MA treatment significantly reversed the expression of apoptosis-related molecules induced by BL-PDT (Fig. 6E, F and Additional file 1: Fig. S4G), increased the cell survival rates (Additional file 1: Fig. S4H), and decreased cell apoptosis rates (Fig. 6G, H and Additional file 1: Fig. S4I). Interestingly, no significant change of the intracellular ROS level was observed (Additional file 1: Fig. S4J-K). Thus, these results indicated that cell autophagy was indeed activated by 450-nm laser/DVDMS-mediated PDT and played a positive role in the process of apoptosis.

### 450-nm laser/DVDMS-mediated PDT induces autophagy via regulating the ROS/PI3K/Akt/mTOR signaling pathway

**Fig. 7:**
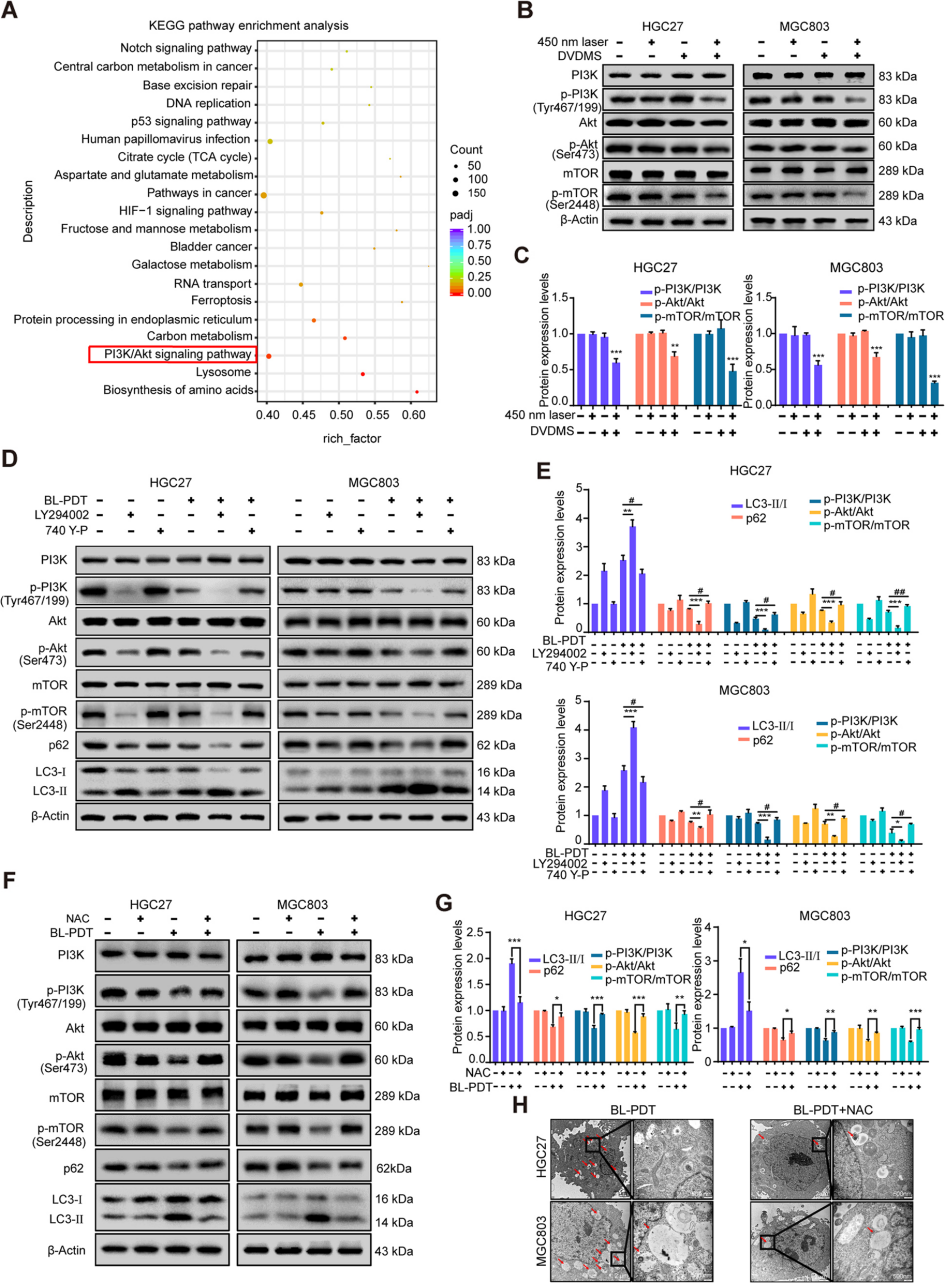
Inhibition of the ROS/PI3K/Akt/mTOR signaling pathway by 450-nm laser/DVDMS-mediated PDT. **A** KEGG pathway enrichment analysis of differentially expressed transcripts in HGC27 cells. **B** Western blotting analysis of the expression levels of pathway-related proteins after the treatment of 24 h. **C** Quantitative analysis of **B** (*n* = 3, mean ± SD). **D** Western blotting analysis of the expression levels of autophagy- and pathway-related proteins after adding inhibitor LY294002 and activator 740 Y-P for 24 h. **E** Quantitative analysis of **D** (*n* = 3, mean ± SD). **F** Western blotting analysis of the expression levels of autophagy- and pathway-related proteins after adding ROS scavenger NAC (incubation 2 h). **G** Quantitative analysis of **F** (*n* = 3, mean ± SD). **H** TEM images of the autophagic vacuoles (indicated by the red arrow) after adding NAC. *p < 0.05, **p < 0.01, and ***p < 0.001

As the classic pathway of autophagy, the PI3K/Akt/mTOR signaling pathway was found to be involved in BL-PDT induced autophagy by KEGG enrichment analysis (Fig. 7A). The protein expression levels of p-PI3K, p-Akt, and p-mTOR in the BL-PDT group were significantly decreased compared with those in the laser or DVDMS alone group, which indicated that the PI3K/Akt/mTOR signaling pathway was inhibited by BL-PDT (Fig. 7B, C and Additional file 1: Fig. S5A). Then, the PI3K inhibitor LY294002 and activator 740 Y-P were used to elucidate the involvement of this pathway. The results showed that the combination of BL-PDT and LY294002 could play a synergistic effect in inhibiting the PI3K/Akt/mTOR signaling pathway and the process of autophagy, while 740 Y-P rescued this effect (Fig. 7D, E and Additional file 1: Fig. S5B-D). Moreover, the expression levels of autophagy-associated proteins were reversed, the PI3K/Akt/mTOR signaling pathway was reactivated, and autophagic vacuoles in the cytoplasm were decreased after adding ROS scavenger NAC (Fig. 7F–H and Additional file 1: Fig. S5B-D). The above results indicated that 450-nm laser/DVDMS-mediated PDT induced autophagy by producing ROS and inhibiting the PI3K/Akt/mTOR signaling pathway.

### 450-nm laser/DVDMS-mediated PDT inhibits tumor growth in vivo

**Fig. 8:**
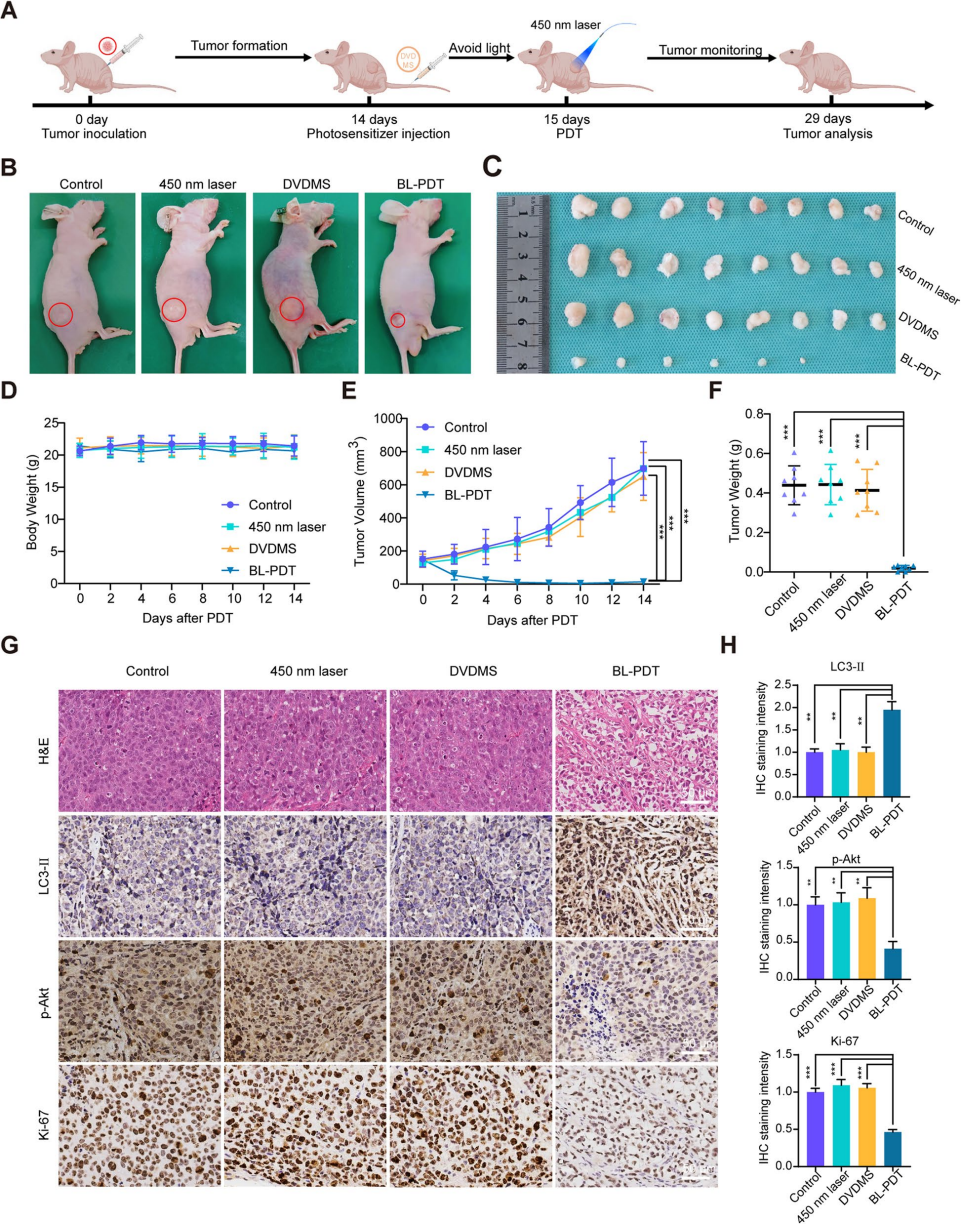
Anticancer effect of 450-nm laser/DVDMS-mediated PDT in vivo. **A** Schematic of time schedule for tumor implantation, DVDMS injection, laser irradiation, and monitoring of tumor growth. **B** Representative photos of tumor-bearing mice after different treatments. **C** Photographs of tumor tissues after different treatments. **D**, **E** Relative body weight and tumor volume changes of mice during different treatments. **F** Tumor weight changes after different treatments. **G** H&E staining and IHC analysis of tumors of the mice after different treatments. Scale bar = 50 μm. **H** Quantitative analysis of **G** (*n* = 3, mean ± SD). *p < 0.05, **p < 0.01, and ***p < 0.001

To explore the antitumor efficiency of BL-PDT in vivo, MGC803 cell subcutaneous xenograft models were constructed in BALB/c nude mice. Typically, xenografted mice received an injection of DVDMS in the caudal vein on day 14, followed by irradiation on the xenografts with a 450-nm laser on the next day (Fig. 8A). The results indicated that tumor growth in the BL-PDT group was significantly inhibited compared with the RL-PDT group or those non-PDT groups (Fig. 8B, C and Additional file 1: Fig. S5F-G). Moreover, the tumor volumes and weights were significantly reduced in the BL-PDT group (Fig. 8E, F and Additional file 1: Fig. S5I-J), while no significant changes were observed in the body weight of nude mice among each group (Fig. 8D and Additional file 1: Fig S5H).

Then, the tumor tissues and important organs of the model mice were analyzed by H&E and IHC. We observed significantly increased expression of LC3-II and decreased expression of p-Akt and Ki-67 in the BL-PDT group (Fig. 8G, H), while no remarkable pathological differences in vital organs (heart, liver, spleen, lung, and kidney) were found among each group (Additional file 1: Fig. S5E). Taken together, the above results indicated that BL-PDT could safely and effectively inhibit the growth of GC in vivo.

## Discussion

**Fig. 9:**
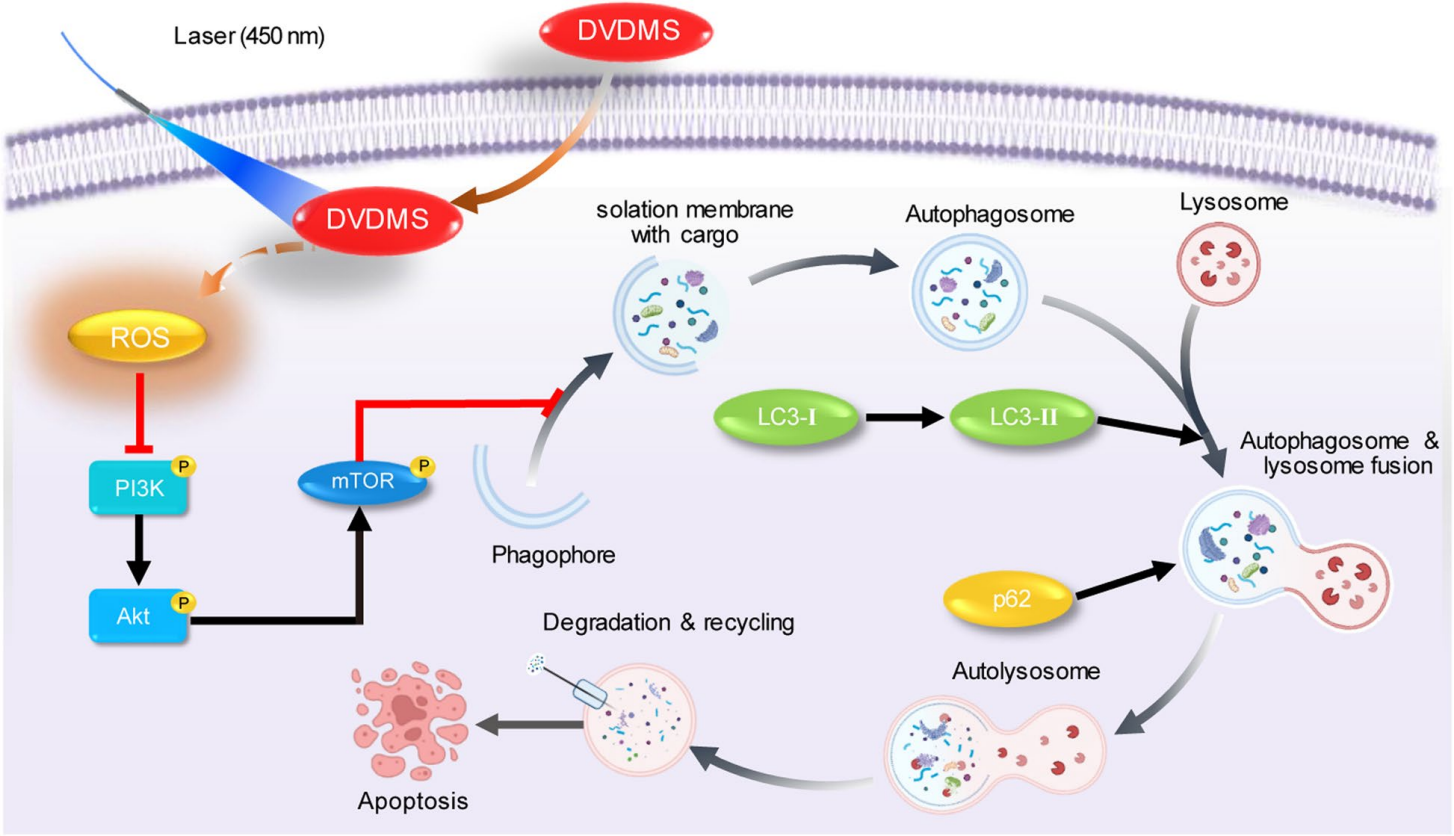
Schematic model of the proposed mechanism of the novel 450-nm laser/DVDMS-mediated PDT in GC cells

Currently, GC is a major global health problem, with an increasing incidence rate and social impact worldwide, especially in Asian countries [29, 30]. For patients with well-differentiated early-stage GC confined to the mucosa or submucosa, a favorable prognosis might be achieved by endoscopic submucosal dissection or suitable surgical operation [31, 32]. Once the tumor infiltrates the muscle layer or metastases, radical operation combined with adjuvant chemoradiotherapy and targeted therapy based on risk factors just can control the patient’s disease as much as possible [33]. As a promising therapy with high efficiency, minimal invasiveness, and few side effects, PDT is considered as an optional diagnostic and therapeutic regimen for GC in many countries, whether patients are in an early or advanced stage [14, 34, 35]. Specifically in our study, the results demonstrated that the 450-nm laser-mediated PDT exerted significant anticancer effects on human GC cells in vitro and in vivo, while it had little toxicity on human gastric mucosal epithelial cells and nude mice. Moreover, we particularly found that BL-PDT might induce remarkable autophagy by regulation of the ROS/PI3K/Akt/mTOR signaling pathway in human GC cells, as illustrated in the schematic diagram in Fig. 9. Hence, in the future, 450-nm laser/DVDMS-mediated PDT will have a broad clinical application in GC treatment.

As reported in previous studies, ROS is one of the main initiators of PDT-induced cancer cell damage [36, 37]. Substantial ROS contributed by PDT may damage cellular lipids, proteins, and DNA; lead to mitochondrial or endoplasmic reticulum (ER) dysfunction; and trigger critical cellular signaling pathways, including apoptosis and autophagy [38]. But in fact, there may be a feedback loop between the accumulation of ROS and the activation of autophagy. As one of the main degradation systems of damaged, denatured, and senescent substances in cells, autophagy could remove not only oxidized/damaged proteins but also bulky ROS-generating organelles (such as mitochondria and peroxisome) to restrict the production and function of ROS [39]. And the study of Scherz-Shouval et al. demonstrated that the low levels of ROS could induce autophagy, which, in turn, degrades catalase, thereby increasing the levels of H_2_O_2_ and ROS, which further induces autophagy [40]. Hence, the accumulation of ROS and activation of autophagy may have a dual role of mutual promotion and inhibition in triggering subsequent cell reaction. In this study, we found that the ROS in GC cells was significantly enhanced after BL-PDT treatment, while the ROS scavenger NAC could reverse this phenotype. Hence, the loss of cell viability should be related to PDT-mediated intracellular ROS production. Nevertheless, the potential mechanism of ROS-induced cell injury and decreased proliferation is not completely clear.

In a study of PDT for human colorectal cancer, large amounts of ROS could induce extensive autophagy via activation of the ROS/JNK signaling pathway, while the anticancer efficiency mediated by verteporfin (VP)-PDT was markedly reduced by the additional use of antioxidants and autophagic inhibitors [41], whereas Mao et al. reported that the combination of carboplatin and 9-hydroxypheophorbide α-PDT could inhibit the epithelial-mesenchymal transformation of human laryngeal cancer cells through ROS-mediated inhibition of the MEK/ERK signaling pathway [42]. In addition, Huang and his colleagues demonstrated that pyropheophorbide-α methyl ester-mediated PDT could suppress the migration/invasion of breast tumor by inhibiting Akt-NF-κB-dependent MMP-9 expression via ROS [43]. In the present study, 450-nm laser/DVDMS-mediated PDT induced autophagy and apoptosis, which might involve the inhibition of the PI3K/Akt/mTOR signaling pathway. However, further studies are still needed to investigate the molecular mechanisms underlying the ROS-mediated regulation of phosphorylation of PI3K.

Effective treatment delivery and exertion in PDT requires the coordination of a light source, photosensitizer, and delivery device appropriate to the target tissue. The wavelength of most PDT light sources used in the clinical setting is 630–850 nm, which has a strong tissue penetration but is easy to attenuate in a liquid environment. As far as photosensitizers are concerned, the light absorption intensity of porphyrin photosensitizers including DVDMS commonly used in clinical practice or experimental study is not quite ideal at 630–880nm [18]. In photodynamic diagnosis and therapy, it is usually necessary to pursue lower light dose and less drug dose to minimize adverse reactions of patients on the premise of PDT effectiveness. Thrillingly, compared with the traditional phototherapeutic laser source, the novel 450-nm laser can more appropriately combine with porphyrin photosensitizer and play a better PDT effect in a variety of tumors under lower light doses and drugs, including gastric cancer, bladder cancer, kidney cancer, and colon cancer (unpublished data). However, it is worth noting that the photodynamic effect is based on the number of photons absorbed by the photosensitizer, which depends on the spectral overlap between the output power of the light source and the optical absorption spectrum of the photosensitizer [28]. Although the novel 450-nm laser is more suitable for the light absorption spectrum of porphyrin photosensitizer, the light dose in clinical photodynamic diagnosis and therapy should be determined by the actual number of photons absorbed, which is helpful to accurately evaluate and compare the PDT effect of different light sources or photosensitizers. In addition, as mentioned above, the 450-nm laser was primarily developed for vaporization and cutting in surgery and also had favorable organizational penetration [26]. If a photosensitizer is given at the same time as a 450-nm laser is used to resect the superficial tumors, the invisible tumor or carcinoma in situ will be killed by the photodynamic effect. We are now performing the single-center clinical trial to evaluate these effects on early gastric cancer and bladder cancer.

More importantly, in addition to producing ROS to directly kill tumor cells, PDT can trigger both humoral and cellular immunity responses by inducing the body to produce a variety of cytokines [44, 45]. Moreover, PDT also has been proven to induce the immunogenic cell death (ICD) of tumor cells through promoting the endogenous antigen release of dying cells and stimulating the activation and infiltration of antigen-specific T cells, as well as their proliferation [46]. In our study, we compared the effects of 450-nm and 630-nm laser-induced PDT in vivo. Interestingly, 450-nm laser-mediated PDT significantly inhibited tumor growth when compared with a 630-nm laser which was known to have a better tissue penetration depth. So, does 450-nm laser-mediated PDT induce more ICD than 630 nm? How does 450-nm laser-mediated PDT simulate the immune response? Further more in-depth studies are needed to solve those questions.

## Conclusions

Collectively, our results showed that the combination of the novel 450-nm laser and photosensitizer DVDMS could generate a stronger photodynamic effect by activating autophagy to trigger apoptosis via regulation of the ROS/PI3K/Akt/mTOR molecular signaling pathway. Our study provides scientific experimental evidence that the novel 450-nm laser-mediated DVDMS-based PDT might be an alternative novel therapeutic strategy for GC in the future.

## Supplementary Information

**Additional file 1: Fig. S1.** Cellular uptake of DVDMS and cytotoxic effects of PDT in GC cells. (A and B) Representative fluorescence images and quantitative analysis of the intracellular uptake of DVDMS after different incubation time points. Scale bar = 10 μm. (n = 3, mean ± SD). (C) Photodynamic effect and cytotoxicity of DVDMS and 5-ALA on cells for 24 h. (n = 3, mean ± SD). (D and F) Colony formation test and quantitative analysis after treating with BL-PDT and RL-PDT. (n = 3, mean ± SD). (E) EdU assay after treating with BL-PDT and RL-PDT. Scale bar = 50 μm. *p < 0.05, **p < 0.01, and ***p < 0.001. **Fig. S2.** Effects of PDT on ROS production in GC cells. (A) Representative fluorescence images of intracellular ROS detection after adding NAC (incubation 2 h). Scale bar = 50 μm. (B) Flow cytometry analysis of the ROS amount in AGS cells after adding NAC (incubation 2 h). (n = 3, mean ± SD). (C) Viability test of GC cells after adding NAC (incubation 2 h). (n = 3, mean ± SD). (D) Colony formation test of GC cells after adding NAC (incubation 2 h). (E) EdU assay of GC cells after adding NAC (incubation 2 h). Scale bar = 50 μm. *p < 0.05, **p < 0.01, and ***p < 0.001. **Fig. S3.** Apoptotic effects of 450 nm laser/DVDMS-mediated PDT on GC cells. (A and B) Heatmap and volcano plot of the DEGs in MGC803 cells between BL-PDT group *vs*. BL group. (C) GO enrichment analysis of the DEGs in MGC803 cells. (D) Flow cytometry analysis of the apoptosis in AGS cells after the treatment of 24 h. (n = 3, mean ± SD). (E) Western blotting analysis of the expression levels of apoptosis related proteins in AGS cells after the treatment of 24 h. (F) Quantitative analysis of (E). (n = 3, mean ± SD). *p < 0.05, **p < 0.01, and ***p < 0.001. **Fig. S4.** 450 nm laser/DVDMS-mediated PDT induced autophagic cell death in GC cells. (A) Representative fluorescence images and quantitative analysis of autophagy dots in AGS cells after the treatment of 24 h. (n = 3, mean ± SD). Scale bar = 10 μm. (B) Typical TEM images of the autophagic vacuoles (indicated by the red arrow) in AGS cells after PDT for 24 h. (C) Western blotting analysis of the expression levels of autophagy related proteins after the treatment of 24 h. (n = 3, mean ± SD). (D) Western blotting analysis of the effect of inhibitor CQ for 4 h on autophagy in AGS cells. (n = 3, mean ± SD). (E) Western blotting analysis of the effect of inhibitor 3-MA for 12 h on autophagy in AGS cells. (n = 3, mean ± SD). (F) Representative fluorescence images and quantitative analysis of autophagy puncta after adding inhibitor 3-MA for 12 h in AGS cells. (n = 3, mean ± SD). Scale bar = 10 μm. (G) Western blotting analysis of the effect of autophagy on BL-PDT inducing apoptosis after adding inhibitor 3-MA in AGS cells. (n = 3, mean ± SD). (H) Viability test of GC cells after adding inhibitor 3-MA. (n = 3, mean ± SD). (I) Flow cytometry analysis of the apoptosis after adding inhibitor 3-MA in AGS cells. (n = 3, mean ± SD). (J) Flow cytometry analysis of the ROS amount in HGC27 cells with different treatments. (K) Quantitative analysis of (J). (n = 3, mean ± SD). *p < 0.05, **p < 0.01, and ***p < 0.001. **Fig. S5.** Effects of 450 nm laser/DVDMS-mediated PDT on ROS/PI3K/Akt/ mTOR signaling pathway and histopathologic change of organs. (A) Western blotting analysis of the expression levels of pathway related proteins in AGS cells after the treatment of 24 h. (n = 3, mean ± SD). (B) TEM images of the autophagic vacuoles (indicated by the red arrow) after adding NAC in AGS cells. (C) Western blotting analysis of the expression levels of autophagy and pathway related proteins after adding LY294002, 740 Y-P and NAC in AGS cells. (D) Quantitative analysis of (C). (n = 3, mean ± SD). (E) H&E staining of vital organs of the mice after different treatments. Scale bar = 200 μm. (F) Representative photos of tumor bearing mice after treating with BL-PDT and RL-PDT. (G) Photographs of tumor tissues after treating with BL-PDT and RL-PDT. (H and I) Relative body weight and tumor volume changes of mice during different treatments. (J) Tumor weight changes after treating with BL-PDT and RL-PDT. *p < 0.05, **p < 0.01, and ***p < 0.001.

**Additional file 2.** Original images of Western blotting results.

## Data Availability

All data supporting the study are included in this published article and its supplementary information files. The datasets generated and used in this study are available from the corresponding author on reasonable request.

## Abbreviations

^1^O_2_: Singlet oxygen
3-MA: 3-Methyladenine
5-ALA: 5-Aminolevulinic acid
BL: Blue laser
CCK-8: Cell Counting Kit-8
CQ: Chloroquine
DCFH-DA: 2′',7′'-Dichlorodihydrofluorescein diacetate
DEGs: Differentially expressed genes
DMPO: 5,5-Dimethyl-1-pyrroline-N-oxide
DVDMS: Sinoporphyrin sodium
EdU: 5-Ethynyl-2′’-deoxyuridine
ER: Endoplasmic reticulum
ESR: Electron spin resonance
GC: Gastric cancer
GSEA: Gene set enrichment analysis
H&E: Hematoxylin and eosin
ICD: Immunogenic cell death
IHC: Immunohistochemistry
LDC: Light dose correction
MFI: Mean fluorescence intensity
MPPa: Pyropheophorbide-α methyl ester
NAC: N-Acetylcysteine
·OH: Hydroxyl radicals
PDT: Photodynamic therapy
PFA: Paraformaldehyde
PI: Propidium iodide
RL: Red laser
ROS: Reactive oxygen species
SD: Standard deviation
TEM: Transmission electron microscopy
TEMP: 2,2,6,6-Tetramethyl-4-piperidone
VP: Verteporfin
