# Supplementary material for: A novel 450-nm laser-mediated sinoporphyrin sodium-based photodynamic therapy induces autophagic cell death in gastric cancer through regulation of the ROS/PI3K/Akt/mTOR signaling pathway
Source: BMC Med. 2022 Dec 8;20:475. doi: 10.1186/s12916-022-02676-8 (PMC9733382; doi:10.1186/s12916-022-02676-8)
Supplement: Supplementary file 1 — Additional file 1: Fig. S1. Cellular uptake of DVDMS and cytotoxic effects of PDT in GC cells. (A and B) Representative fluorescence images and quantitative analysis of the intracellular uptake of DVDMS after different incubation time points. Scale bar = 10 μm. (n = 3, mean ± SD). (C) Photodynamic effect and cytotoxicity of DVDMS and 5-ALA on cells for 24 h. (n = 3, mean ± SD). (D and F) Colony formation test and quantitative analysis after treating with BL-PDT and RL-PDT. (n = 3, mean ± SD). (E) EdU assay after treating with BL-PDT and RL-PDT. Scale bar = 50 μm. *p < 0.05, **p < 0.01, and ***p < 0.001. Fig. S2. Effects of PDT on ROS production in GC cells. (A) Representative fluorescence images of intracellular ROS detection after adding NAC (incubation 2 h). Scale bar = 50 μm. (B) Flow cytometry analysis of the ROS amount in AGS cells after adding NAC (incubation 2 h). (n = 3, mean ± SD). (C) Viability test of GC cells after adding NAC (incubation 2 h). (n = 3, mean ± SD). (D) Colony formation test of GC cells after adding NAC (incubation 2 h). (E) EdU assay of GC cells after adding NAC (incubation 2 h). Scale bar = 50 μm. *p < 0.05, **p < 0.01, and ***p < 0.001. Fig. S3. Apoptotic effects of 450 nm laser/DVDMS-mediated PDT on GC cells. (A and B) Heatmap and volcano plot of the DEGs in MGC803 cells between BL-PDT group vs. BL group. (C) GO enrichment analysis of the DEGs in MGC803 cells. (D) Flow cytometry analysis of the apoptosis in AGS cells after the treatment of 24 h. (n = 3, mean ± SD). (E) Western blotting analysis of the expression levels of apoptosis related proteins in AGS cells after the treatment of 24 h. (F) Quantitative analysis of (E). (n = 3, mean ± SD). *p < 0.05, **p < 0.01, and ***p < 0.001. Fig. S4. 450 nm laser/DVDMS-mediated PDT induced autophagic cell death in GC cells. (A) Representative fluorescence images and quantitative analysis of autophagy dots in AGS cells after the treatment of 24 h. (n = 3, mean ± SD). Scale bar = 10 μm. (B) Typica [file 12916_2022_2676_MOESM1_ESM.zip › Additional File 1R3.docx]

**Additional file 1: Fig. S1-S5.** Fig. S1: Cellular uptake of DVDMS and cytotoxic effects of PDT in GC cells. (A and B) Representative fluorescence images and quantitative analysis of the intracellular uptake of DVDMS after different incubation time points. Scale bar = 10 μm. (n = 3, mean ± SD). (C) Photodynamic effect and cytotoxicity of DVDMS and 5-ALA on cells for 24 h. (n = 3, mean ± SD). (D and F) Colony formation test and quantitative analysis after treating with BL-PDT and RL-PDT. (n = 3, mean ± SD). (E) EdU assay after treating with BL-PDT and RL-PDT. Scale bar = 50 μm. * *p* < 0.05; ** *p* < 0.01 and *** *p* < 0.001. Fig. S2: Effects of PDT on ROS production in GC cells. (A) Representative fluorescence images of intracellular ROS detection after adding NAC (incubation 2 h). Scale bar = 50 μm. (B) Flow cytometry analysis of the ROS amount in AGS cells after adding NAC (incubation 2 h). (n = 3, mean ± SD). (C) Viability test of GC cells after adding NAC (incubation 2 h). (n = 3, mean ± SD). (D) Colony formation test of GC cells after adding NAC (incubation 2 h). (E) EdU assay of GC cells after adding NAC (incubation 2 h). Scale bar = 50 μm. * *p* < 0.05; ** *p* < 0.01 and *** *p* < 0.001. Fig. S3: Apoptotic effects of 450 nm laser/DVDMS-mediated PDT on GC cells. (A and B) Heatmap and volcano plot of the DEGs in MGC803 cells between BL-PDT group *vs*. BL group. (C) GO enrichment analysis of the DEGs in MGC803 cells. (D) Flow cytometry analysis of the apoptosis in AGS cells after the treatment of 24 h. (n = 3, mean ± SD). (E) Western blotting analysis of the expression levels of apoptosis related proteins in AGS cells after the treatment of 24 h. (F) Quantitative analysis of (E). (n = 3, mean ± SD). * *p* < 0.05; ** *p* < 0.01 and *** *p* < 0.001. Fig. S4**:** 450 nm laser/DVDMS-mediated PDT induced autophagic cell death in GC cells. (A) Representative fluorescence images and quantitative analysis of autophagy dots in AGS cells after the treatment of 24 h. (n = 3, mean ± SD). Scale bar = 10 μm. (B) Typical TEM images of the autophagic vacuoles (indicated by the red arrow) in AGS cells after PDT for 24 h. (C) Western blotting analysis of the expression levels of autophagy related proteins after the treatment of 24 h. (n = 3, mean ± SD). (D) Western blotting analysis of the effect of inhibitor CQ for 4 h on autophagy in AGS cells. (n = 3, mean ± SD). (E) Western blotting analysis of the effect of inhibitor 3-MA for 12 h on autophagy in AGS cells. (n = 3, mean ± SD). (F) Representative fluorescence images and quantitative analysis of autophagy puncta after adding inhibitor 3-MA for 12 h in AGS cells. (n = 3, mean ± SD). Scale bar = 10 μm. (G) Western blotting analysis of the effect of autophagy on BL-PDT inducing apoptosis after adding inhibitor 3-MA in AGS cells. (n = 3, mean ± SD). (H) Viability test of GC cells after adding inhibitor 3-MA. (n = 3, mean ± SD). (I) Flow cytometry analysis of the apoptosis after adding inhibitor 3-MA in AGS cells. (n = 3, mean ± SD). (J) Flow cytometry analysis of the ROS amount in HGC27 cells with different treatments. (K) Quantitative analysis of (J). (n = 3, mean ± SD). * *p* < 0.05; ** *p* < 0.01 and *** *p* < 0.001. Fig. S5: Effects of 450 nm laser/DVDMS-mediated PDT on ROS/PI3K/Akt/ mTOR signaling pathway and histopathologic change of organs. (A) Western blotting analysis of the expression levels of pathway related proteins in AGS cells after the treatment of 24 h. (n = 3, mean ± SD). (B) TEM images of the autophagic vacuoles (indicated by the red arrow) after adding NAC in AGS cells. (C) Western blotting analysis of the expression levels of autophagy and pathway related proteins after adding LY294002, 740 Y-P and NAC in AGS cells. (D) Quantitative analysis of (C). (n = 3, mean ± SD). (E) H&E staining of vital organs of the mice after different treatments. Scale bar = 200 μm. (F) Representative photos of tumor bearing mice after treating with BL-PDT and RL-PDT. (G) Photographs of tumor tissues after treating with BL-PDT and RL-PDT. (H and I) Relative body weight and tumor volume changes of mice during different treatments. (J) Tumor weight changes after treating with BL-PDT and RL-PDT. * *p* < 0.05; ** *p* < 0.01 and *** *p* < 0.001.
