# Supplementary figures and images for: A novel 450-nm laser-mediated sinoporphyrin sodium-based photodynamic therapy induces autophagic cell death in gastric cancer through regulation of the ROS/PI3K/Akt/mTOR signaling pathway
Source: BMC Med. 2022 Dec 8;20:475. doi: 10.1186/s12916-022-02676-8 (PMC9733382; doi:10.1186/s12916-022-02676-8)

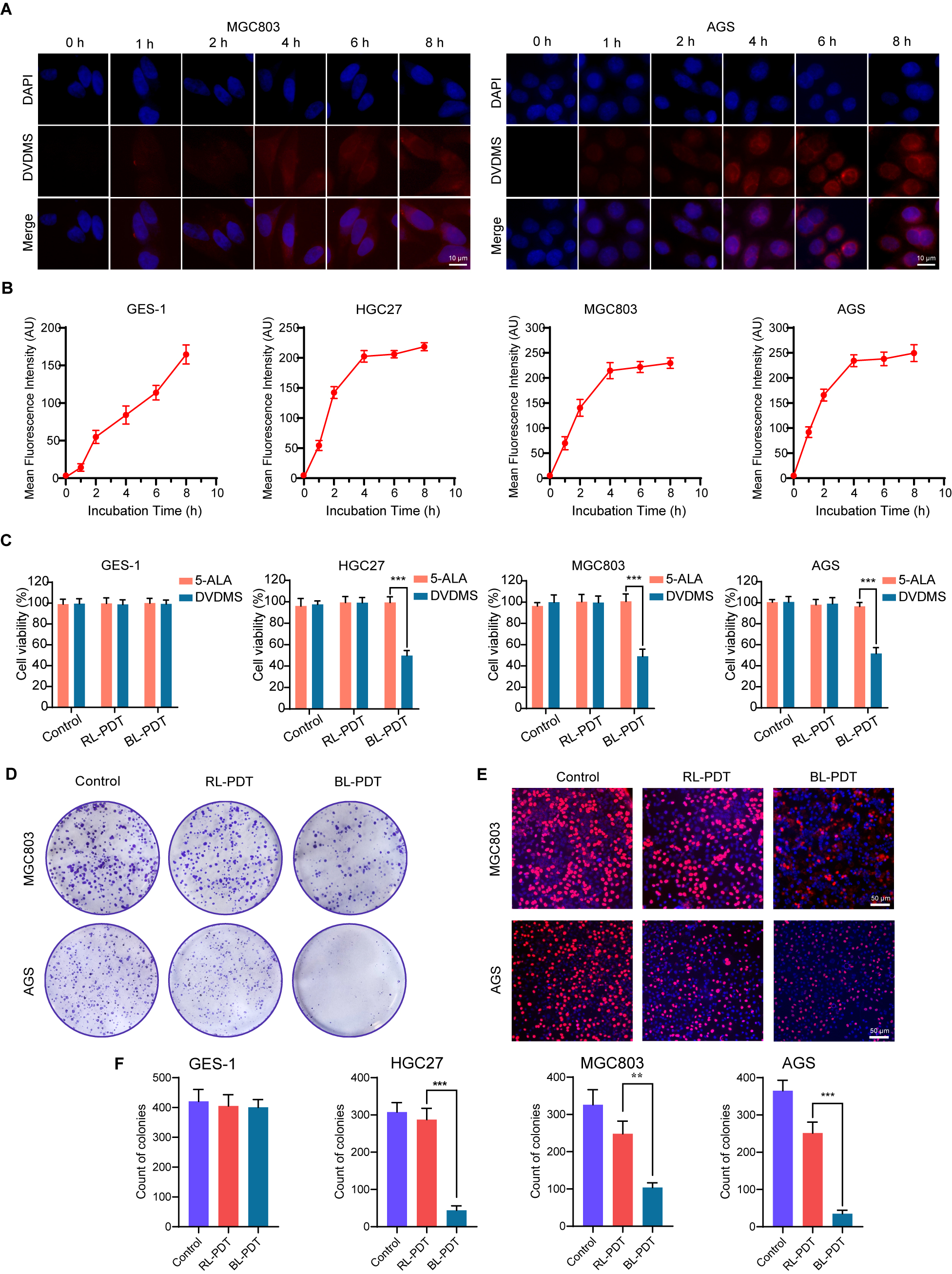

Supplement: Supplementary file 1 — Additional file 1: Fig. S1. Cellular uptake of DVDMS and cytotoxic effects of PDT in GC cells. (A and B) Representative fluorescence images and quantitative analysis of the intracellular uptake of DVDMS after different incubation time points. Scale bar = 10 μm. (n = 3, mean ± SD). (C) Photodynamic effect and cytotoxicity of DVDMS and 5-ALA on cells for 24 h. (n = 3, mean ± SD). (D and F) Colony formation test and quantitative analysis after treating with BL-PDT and RL-PDT. (n = 3, mean ± SD). (E) EdU assay after treating with BL-PDT and RL-PDT. Scale bar = 50 μm. *p < 0.05, **p < 0.01, and ***p < 0.001. Fig. S2. Effects of PDT on ROS production in GC cells. (A) Representative fluorescence images of intracellular ROS detection after adding NAC (incubation 2 h). Scale bar = 50 μm. (B) Flow cytometry analysis of the ROS amount in AGS cells after adding NAC (incubation 2 h). (n = 3, mean ± SD). (C) Viability test of GC cells after adding NAC (incubation 2 h). (n = 3, mean ± SD). (D) Colony formation test of GC cells after adding NAC (incubation 2 h). (E) EdU assay of GC cells after adding NAC (incubation 2 h). Scale bar = 50 μm. *p < 0.05, **p < 0.01, and ***p < 0.001. Fig. S3. Apoptotic effects of 450 nm laser/DVDMS-mediated PDT on GC cells. (A and B) Heatmap and volcano plot of the DEGs in MGC803 cells between BL-PDT group vs. BL group. (C) GO enrichment analysis of the DEGs in MGC803 cells. (D) Flow cytometry analysis of the apoptosis in AGS cells after the treatment of 24 h. (n = 3, mean ± SD). (E) Western blotting analysis of the expression levels of apoptosis related proteins in AGS cells after the treatment of 24 h. (F) Quantitative analysis of (E). (n = 3, mean ± SD). *p < 0.05, **p < 0.01, and ***p < 0.001. Fig. S4. 450 nm laser/DVDMS-mediated PDT induced autophagic cell death in GC cells. (A) Representative fluorescence images and quantitative analysis of autophagy dots in AGS cells after the treatment of 24 h. (n = 3, mean ± SD). Scale bar = 10 μm. (B) Typica [file 12916_2022_2676_MOESM1_ESM.zip › Fig. S1R3.jpg]

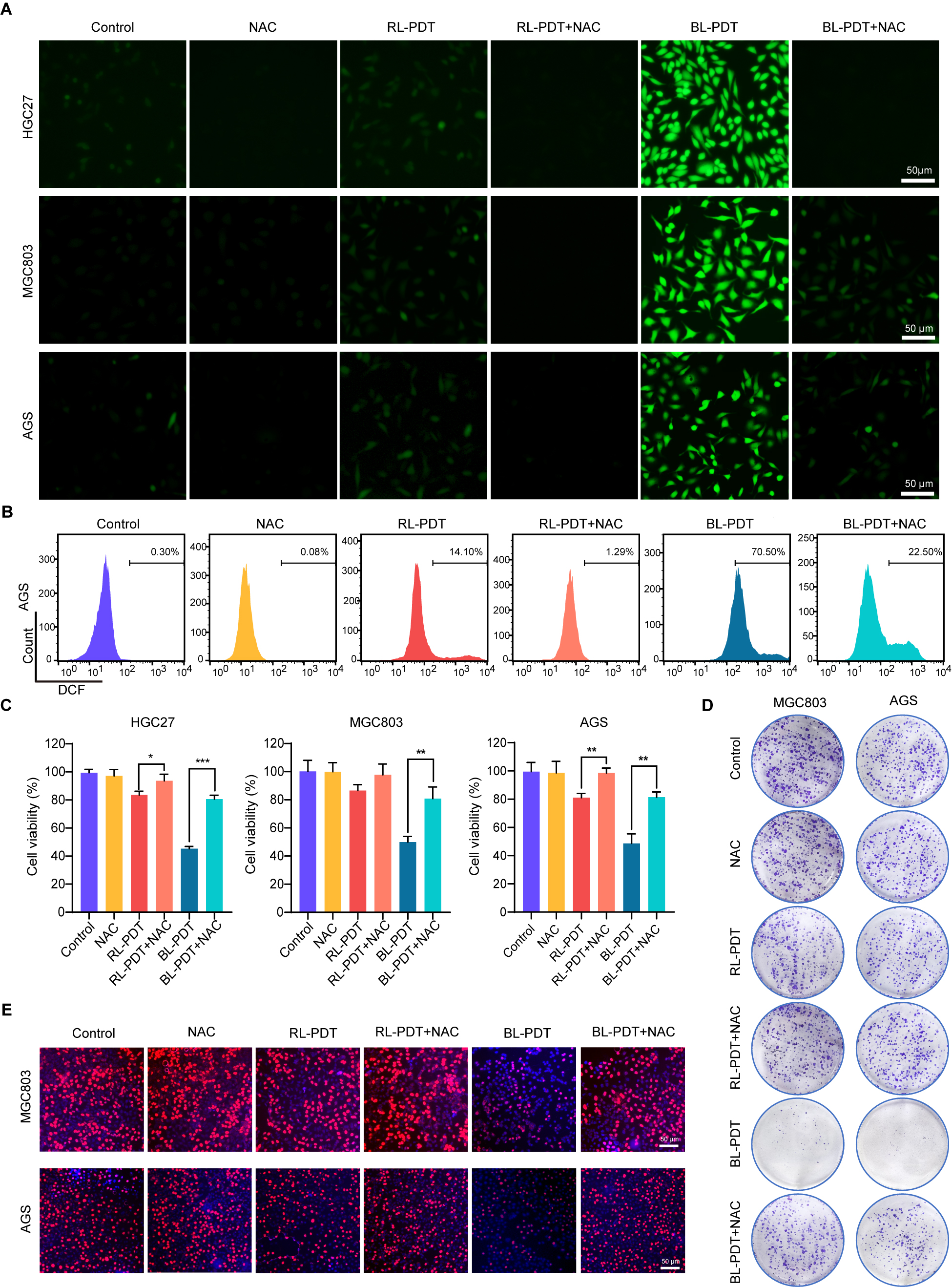

Supplement: Supplementary file 1 — Additional file 1: Fig. S1. Cellular uptake of DVDMS and cytotoxic effects of PDT in GC cells. (A and B) Representative fluorescence images and quantitative analysis of the intracellular uptake of DVDMS after different incubation time points. Scale bar = 10 μm. (n = 3, mean ± SD). (C) Photodynamic effect and cytotoxicity of DVDMS and 5-ALA on cells for 24 h. (n = 3, mean ± SD). (D and F) Colony formation test and quantitative analysis after treating with BL-PDT and RL-PDT. (n = 3, mean ± SD). (E) EdU assay after treating with BL-PDT and RL-PDT. Scale bar = 50 μm. *p < 0.05, **p < 0.01, and ***p < 0.001. Fig. S2. Effects of PDT on ROS production in GC cells. (A) Representative fluorescence images of intracellular ROS detection after adding NAC (incubation 2 h). Scale bar = 50 μm. (B) Flow cytometry analysis of the ROS amount in AGS cells after adding NAC (incubation 2 h). (n = 3, mean ± SD). (C) Viability test of GC cells after adding NAC (incubation 2 h). (n = 3, mean ± SD). (D) Colony formation test of GC cells after adding NAC (incubation 2 h). (E) EdU assay of GC cells after adding NAC (incubation 2 h). Scale bar = 50 μm. *p < 0.05, **p < 0.01, and ***p < 0.001. Fig. S3. Apoptotic effects of 450 nm laser/DVDMS-mediated PDT on GC cells. (A and B) Heatmap and volcano plot of the DEGs in MGC803 cells between BL-PDT group vs. BL group. (C) GO enrichment analysis of the DEGs in MGC803 cells. (D) Flow cytometry analysis of the apoptosis in AGS cells after the treatment of 24 h. (n = 3, mean ± SD). (E) Western blotting analysis of the expression levels of apoptosis related proteins in AGS cells after the treatment of 24 h. (F) Quantitative analysis of (E). (n = 3, mean ± SD). *p < 0.05, **p < 0.01, and ***p < 0.001. Fig. S4. 450 nm laser/DVDMS-mediated PDT induced autophagic cell death in GC cells. (A) Representative fluorescence images and quantitative analysis of autophagy dots in AGS cells after the treatment of 24 h. (n = 3, mean ± SD). Scale bar = 10 μm. (B) Typica [file 12916_2022_2676_MOESM1_ESM.zip › Fig. S2R3.jpg]

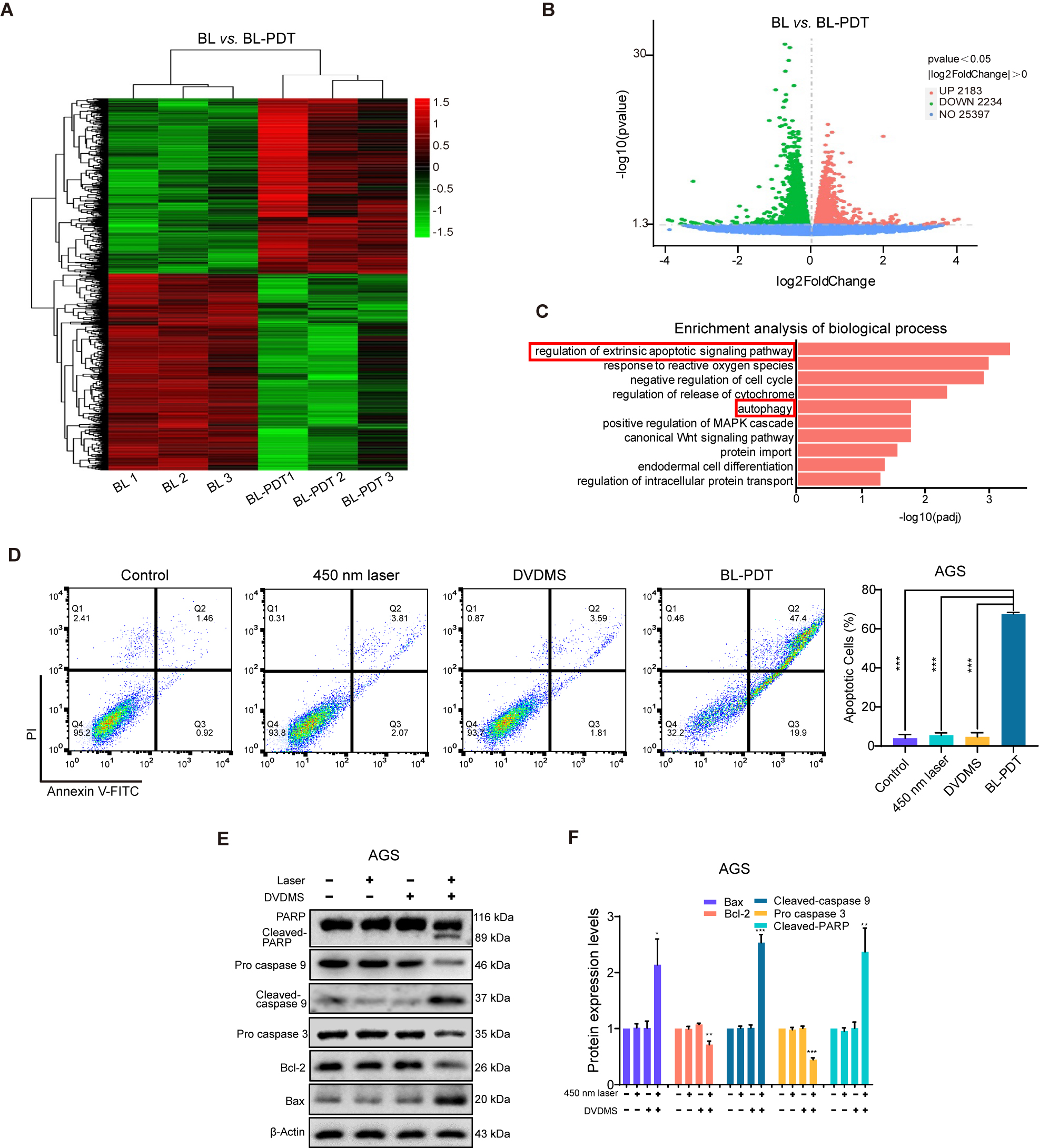

Supplement: Supplementary file 1 — Additional file 1: Fig. S1. Cellular uptake of DVDMS and cytotoxic effects of PDT in GC cells. (A and B) Representative fluorescence images and quantitative analysis of the intracellular uptake of DVDMS after different incubation time points. Scale bar = 10 μm. (n = 3, mean ± SD). (C) Photodynamic effect and cytotoxicity of DVDMS and 5-ALA on cells for 24 h. (n = 3, mean ± SD). (D and F) Colony formation test and quantitative analysis after treating with BL-PDT and RL-PDT. (n = 3, mean ± SD). (E) EdU assay after treating with BL-PDT and RL-PDT. Scale bar = 50 μm. *p < 0.05, **p < 0.01, and ***p < 0.001. Fig. S2. Effects of PDT on ROS production in GC cells. (A) Representative fluorescence images of intracellular ROS detection after adding NAC (incubation 2 h). Scale bar = 50 μm. (B) Flow cytometry analysis of the ROS amount in AGS cells after adding NAC (incubation 2 h). (n = 3, mean ± SD). (C) Viability test of GC cells after adding NAC (incubation 2 h). (n = 3, mean ± SD). (D) Colony formation test of GC cells after adding NAC (incubation 2 h). (E) EdU assay of GC cells after adding NAC (incubation 2 h). Scale bar = 50 μm. *p < 0.05, **p < 0.01, and ***p < 0.001. Fig. S3. Apoptotic effects of 450 nm laser/DVDMS-mediated PDT on GC cells. (A and B) Heatmap and volcano plot of the DEGs in MGC803 cells between BL-PDT group vs. BL group. (C) GO enrichment analysis of the DEGs in MGC803 cells. (D) Flow cytometry analysis of the apoptosis in AGS cells after the treatment of 24 h. (n = 3, mean ± SD). (E) Western blotting analysis of the expression levels of apoptosis related proteins in AGS cells after the treatment of 24 h. (F) Quantitative analysis of (E). (n = 3, mean ± SD). *p < 0.05, **p < 0.01, and ***p < 0.001. Fig. S4. 450 nm laser/DVDMS-mediated PDT induced autophagic cell death in GC cells. (A) Representative fluorescence images and quantitative analysis of autophagy dots in AGS cells after the treatment of 24 h. (n = 3, mean ± SD). Scale bar = 10 μm. (B) Typica [file 12916_2022_2676_MOESM1_ESM.zip › Fig. S3R3.jpg]

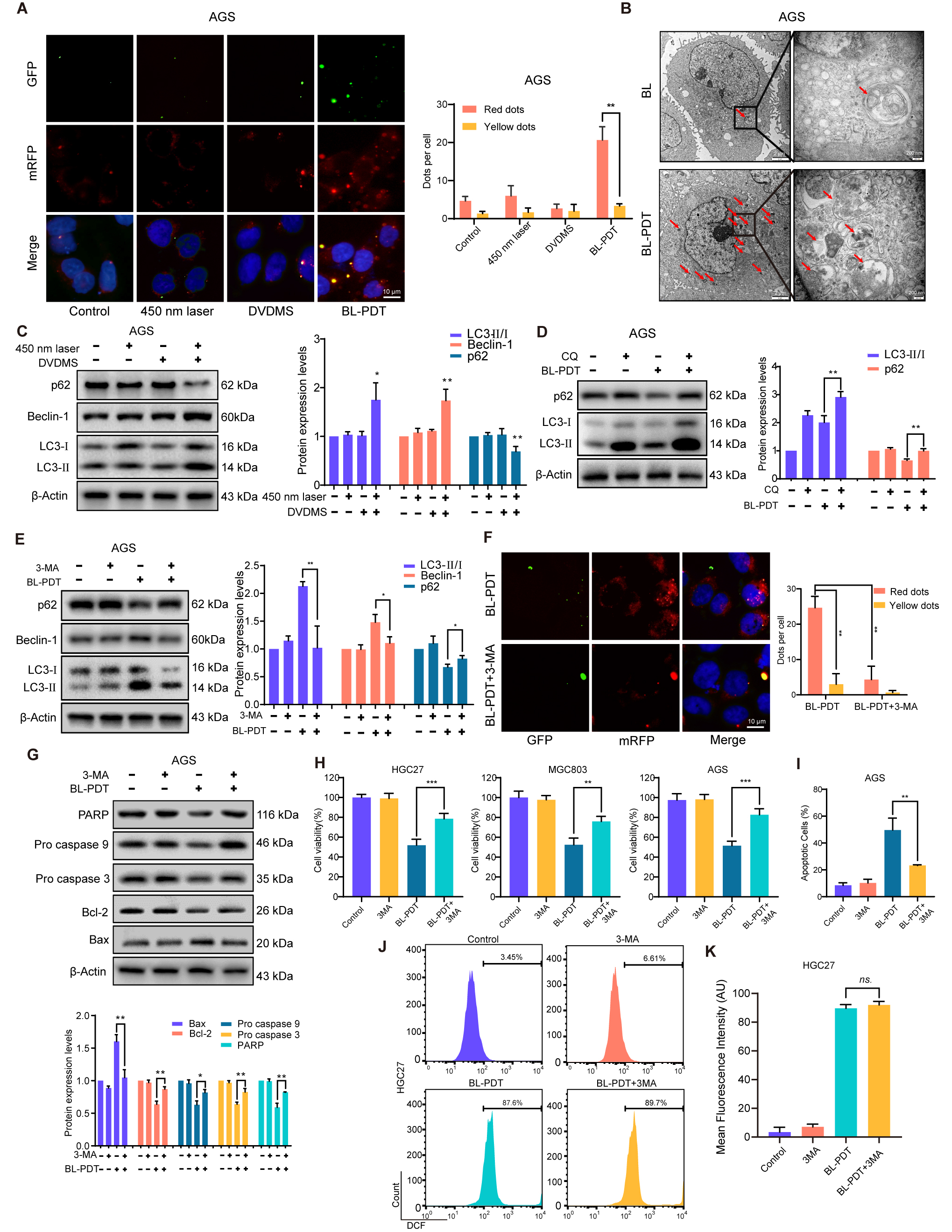

Supplement: Supplementary file 1 — Additional file 1: Fig. S1. Cellular uptake of DVDMS and cytotoxic effects of PDT in GC cells. (A and B) Representative fluorescence images and quantitative analysis of the intracellular uptake of DVDMS after different incubation time points. Scale bar = 10 μm. (n = 3, mean ± SD). (C) Photodynamic effect and cytotoxicity of DVDMS and 5-ALA on cells for 24 h. (n = 3, mean ± SD). (D and F) Colony formation test and quantitative analysis after treating with BL-PDT and RL-PDT. (n = 3, mean ± SD). (E) EdU assay after treating with BL-PDT and RL-PDT. Scale bar = 50 μm. *p < 0.05, **p < 0.01, and ***p < 0.001. Fig. S2. Effects of PDT on ROS production in GC cells. (A) Representative fluorescence images of intracellular ROS detection after adding NAC (incubation 2 h). Scale bar = 50 μm. (B) Flow cytometry analysis of the ROS amount in AGS cells after adding NAC (incubation 2 h). (n = 3, mean ± SD). (C) Viability test of GC cells after adding NAC (incubation 2 h). (n = 3, mean ± SD). (D) Colony formation test of GC cells after adding NAC (incubation 2 h). (E) EdU assay of GC cells after adding NAC (incubation 2 h). Scale bar = 50 μm. *p < 0.05, **p < 0.01, and ***p < 0.001. Fig. S3. Apoptotic effects of 450 nm laser/DVDMS-mediated PDT on GC cells. (A and B) Heatmap and volcano plot of the DEGs in MGC803 cells between BL-PDT group vs. BL group. (C) GO enrichment analysis of the DEGs in MGC803 cells. (D) Flow cytometry analysis of the apoptosis in AGS cells after the treatment of 24 h. (n = 3, mean ± SD). (E) Western blotting analysis of the expression levels of apoptosis related proteins in AGS cells after the treatment of 24 h. (F) Quantitative analysis of (E). (n = 3, mean ± SD). *p < 0.05, **p < 0.01, and ***p < 0.001. Fig. S4. 450 nm laser/DVDMS-mediated PDT induced autophagic cell death in GC cells. (A) Representative fluorescence images and quantitative analysis of autophagy dots in AGS cells after the treatment of 24 h. (n = 3, mean ± SD). Scale bar = 10 μm. (B) Typica [file 12916_2022_2676_MOESM1_ESM.zip › Fig. S4R3.jpg]

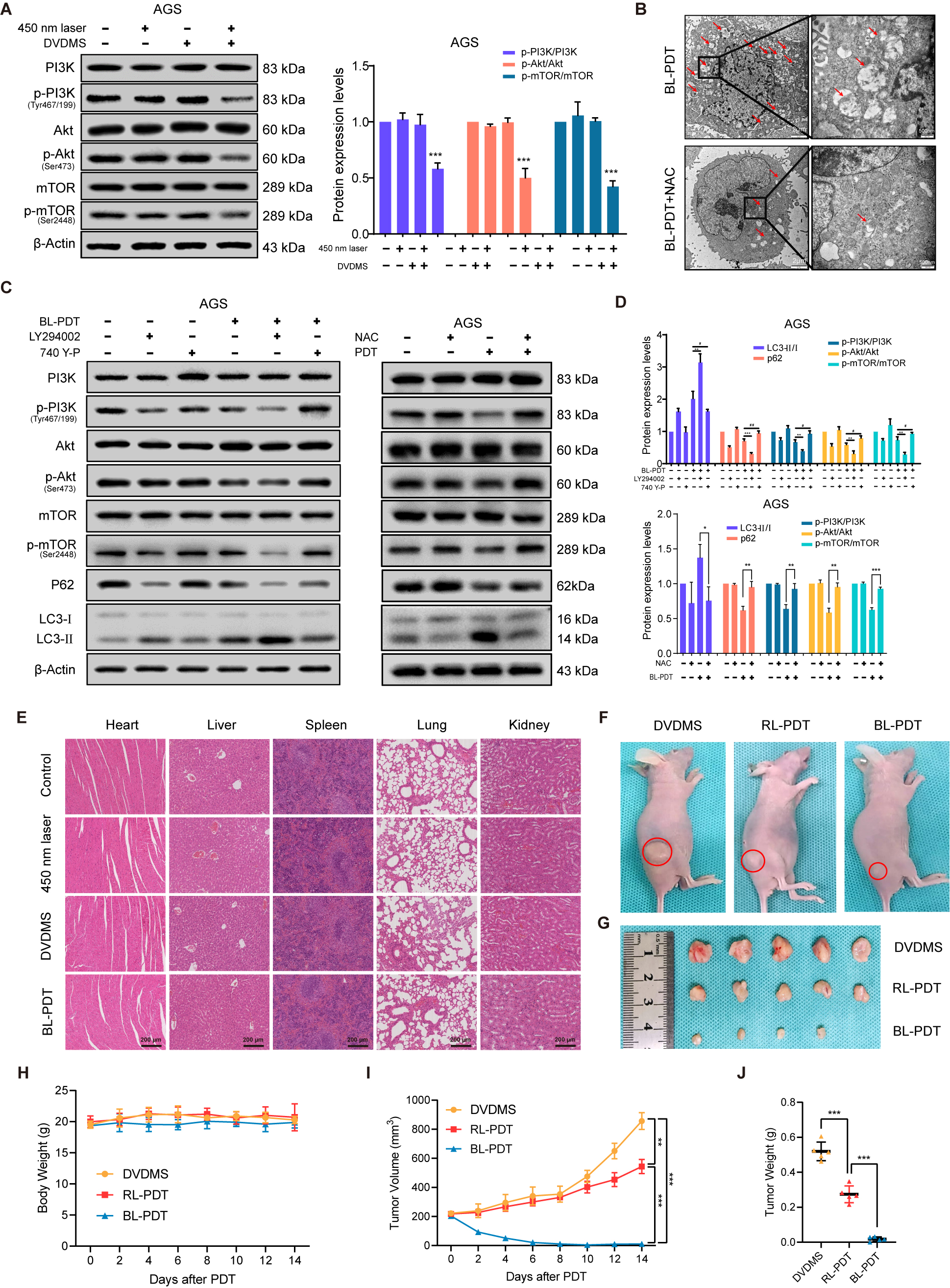

Supplement: Supplementary file 1 — Additional file 1: Fig. S1. Cellular uptake of DVDMS and cytotoxic effects of PDT in GC cells. (A and B) Representative fluorescence images and quantitative analysis of the intracellular uptake of DVDMS after different incubation time points. Scale bar = 10 μm. (n = 3, mean ± SD). (C) Photodynamic effect and cytotoxicity of DVDMS and 5-ALA on cells for 24 h. (n = 3, mean ± SD). (D and F) Colony formation test and quantitative analysis after treating with BL-PDT and RL-PDT. (n = 3, mean ± SD). (E) EdU assay after treating with BL-PDT and RL-PDT. Scale bar = 50 μm. *p < 0.05, **p < 0.01, and ***p < 0.001. Fig. S2. Effects of PDT on ROS production in GC cells. (A) Representative fluorescence images of intracellular ROS detection after adding NAC (incubation 2 h). Scale bar = 50 μm. (B) Flow cytometry analysis of the ROS amount in AGS cells after adding NAC (incubation 2 h). (n = 3, mean ± SD). (C) Viability test of GC cells after adding NAC (incubation 2 h). (n = 3, mean ± SD). (D) Colony formation test of GC cells after adding NAC (incubation 2 h). (E) EdU assay of GC cells after adding NAC (incubation 2 h). Scale bar = 50 μm. *p < 0.05, **p < 0.01, and ***p < 0.001. Fig. S3. Apoptotic effects of 450 nm laser/DVDMS-mediated PDT on GC cells. (A and B) Heatmap and volcano plot of the DEGs in MGC803 cells between BL-PDT group vs. BL group. (C) GO enrichment analysis of the DEGs in MGC803 cells. (D) Flow cytometry analysis of the apoptosis in AGS cells after the treatment of 24 h. (n = 3, mean ± SD). (E) Western blotting analysis of the expression levels of apoptosis related proteins in AGS cells after the treatment of 24 h. (F) Quantitative analysis of (E). (n = 3, mean ± SD). *p < 0.05, **p < 0.01, and ***p < 0.001. Fig. S4. 450 nm laser/DVDMS-mediated PDT induced autophagic cell death in GC cells. (A) Representative fluorescence images and quantitative analysis of autophagy dots in AGS cells after the treatment of 24 h. (n = 3, mean ± SD). Scale bar = 10 μm. (B) Typica [file 12916_2022_2676_MOESM1_ESM.zip › Fig. S5R3.jpg]
