# Supplementary material for: A novel 450-nm laser-mediated sinoporphyrin sodium-based photodynamic therapy induces autophagic cell death in gastric cancer through regulation of the ROS/PI3K/Akt/mTOR signaling pathway
Source: BMC Med. 2022 Dec 8;20:475. doi: 10.1186/s12916-022-02676-8 (PMC9733382; doi:10.1186/s12916-022-02676-8)
Supplement: Supplementary file 2 — Additional file 2. Original images of Western blotting results. [file 12916_2022_2676_MOESM2_ESM.docx]

**Additional File 2:** Original images of Western blotting results.


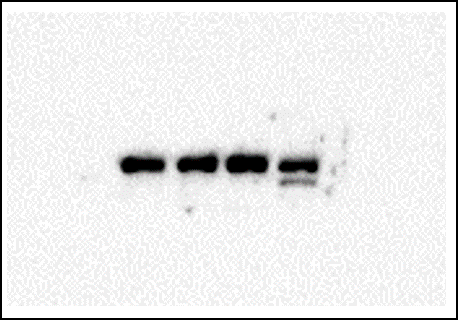
1. Original images of Figure 4G and S3E.


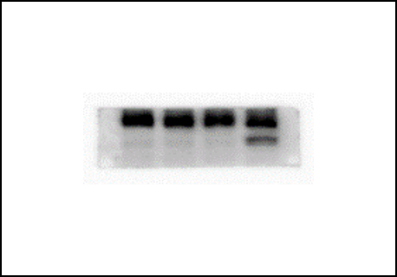

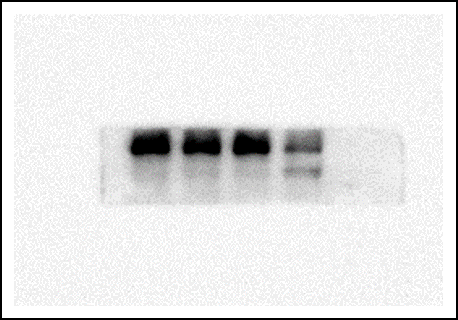

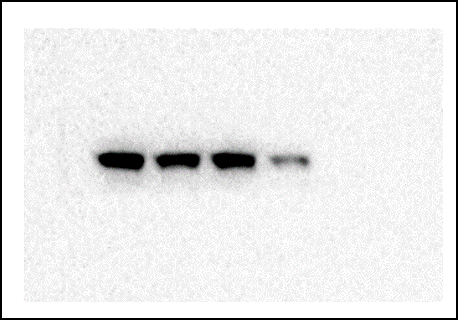

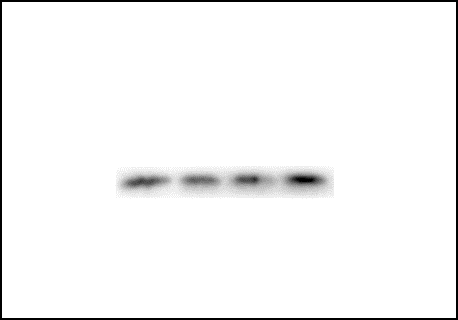

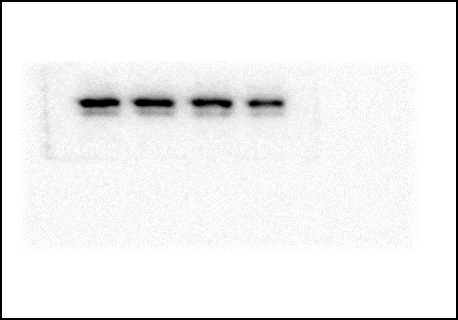

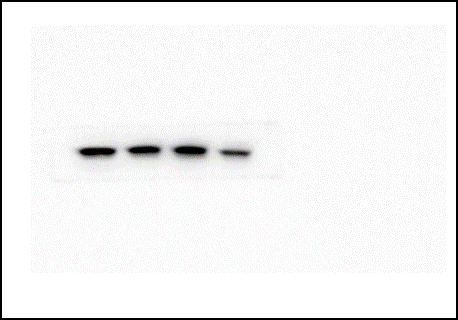

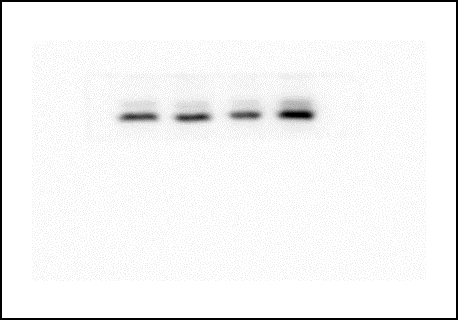

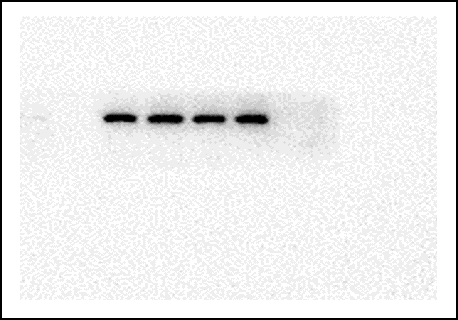


AGS

HGC27

MGC803


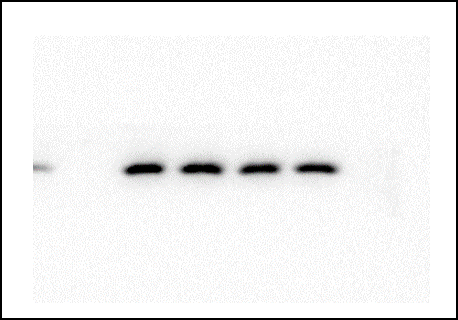

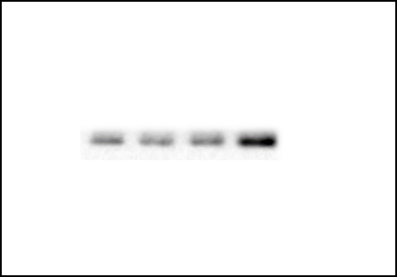

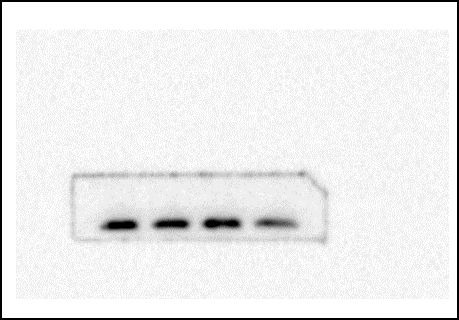

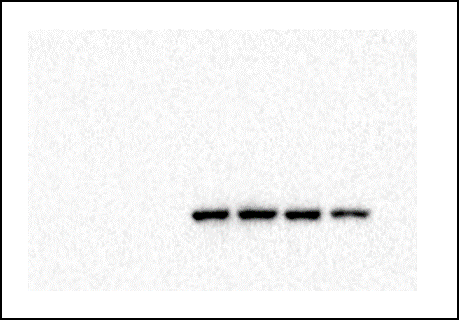

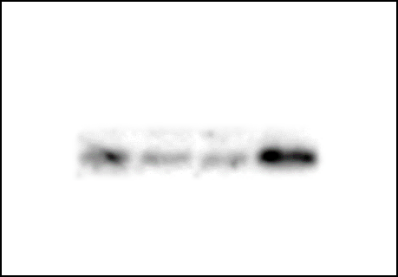

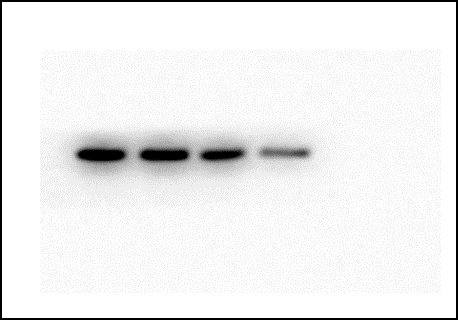

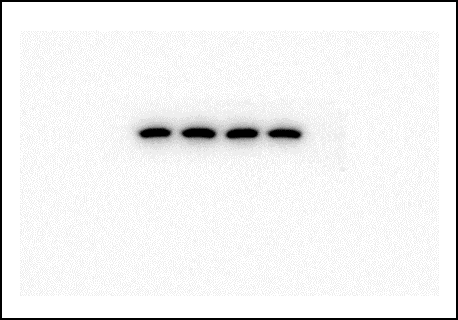

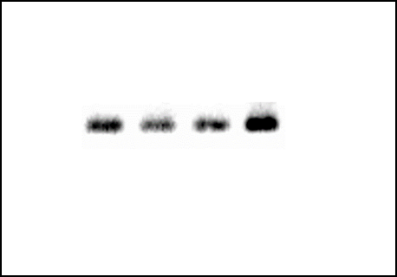

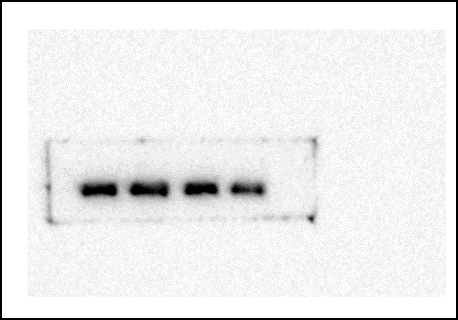

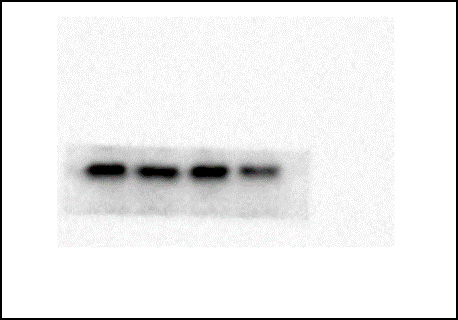

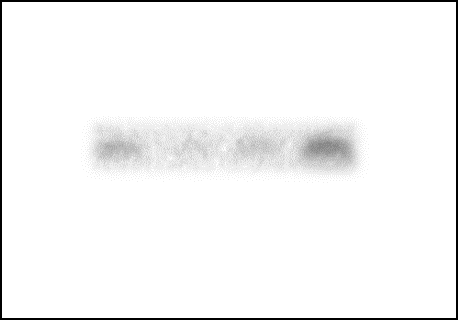

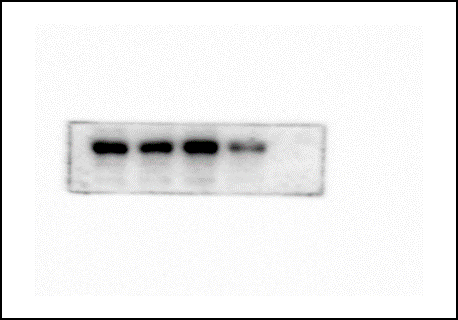


PARP

Bax

Pro caspase 9

β-Actin

Bcl-2

Cleaved- caspase 9

Pro caspase 3

Pro caspase 3

Cleaved- caspase 9

Bcl-2

β-Actin

Pro caspase 9

Bax

PARP

Cleaved- caspase 9

Bcl-2

Pro caspase 3

Bax

β-Actin

Pro caspase 9

PARP


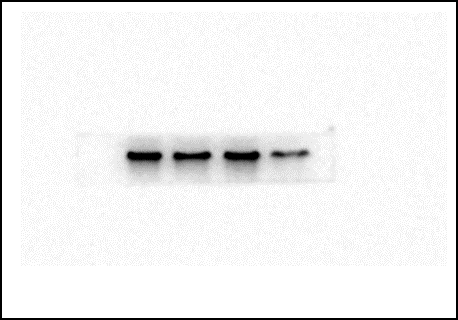

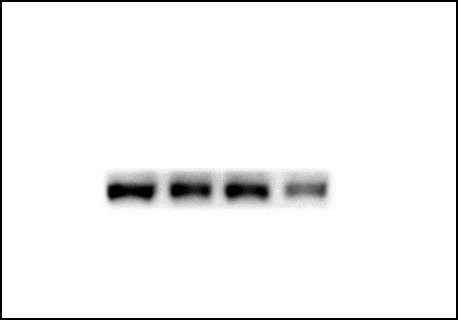
2. Original images of Figure 5D and S4C.


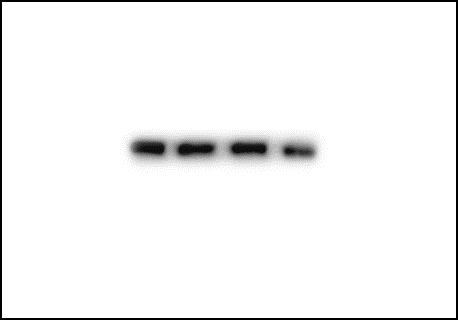


HGC27

MGC803

AGS

p62

p62

p62

Beclin-1

β-Actin

LC3

Beclin-1

β-Actin

LC3

Beclin-1

β-Actin

LC3


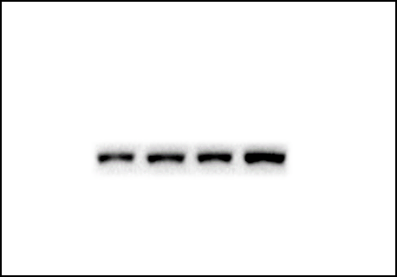

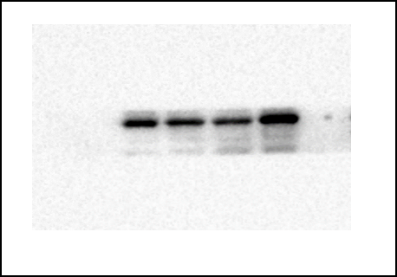

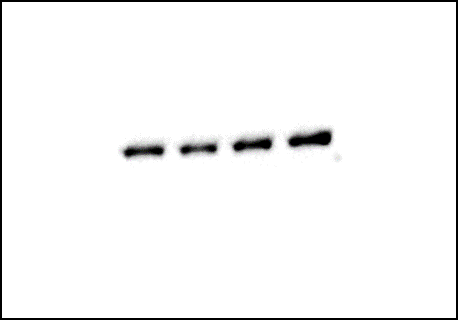


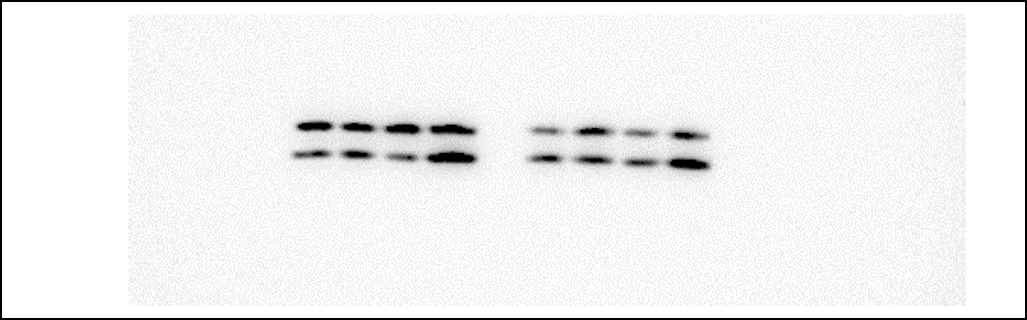

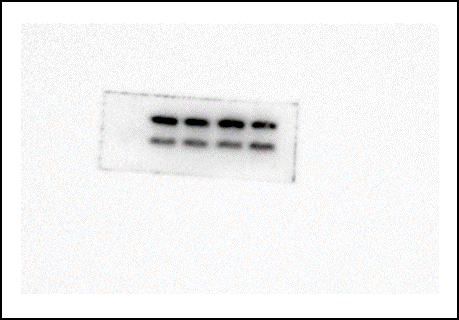


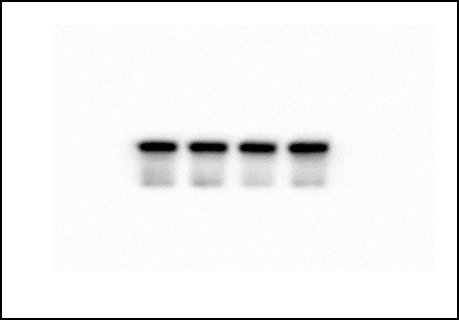

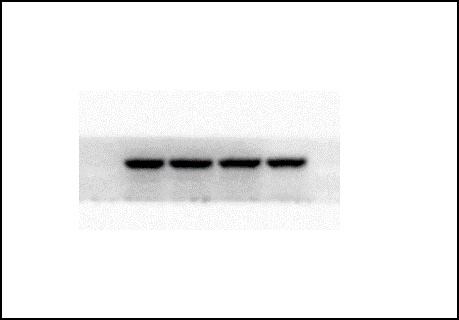

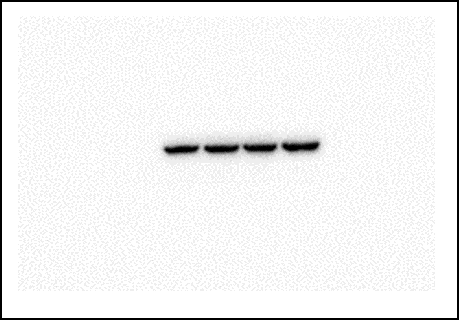


3. Original images of Figure 5F and S4D.


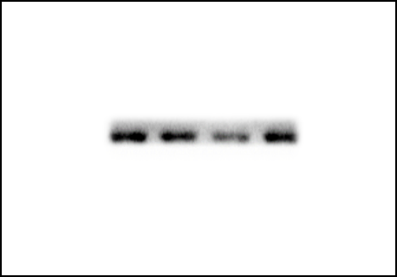

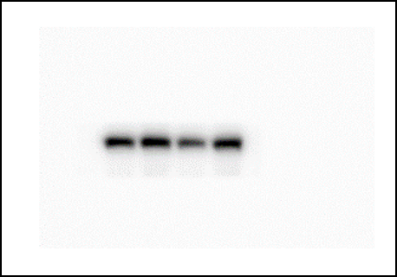

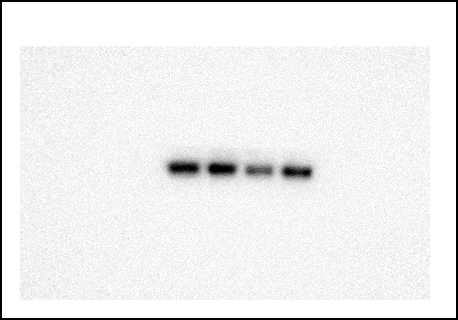


AGS

MGC803

HGC27

p62

p62

p62


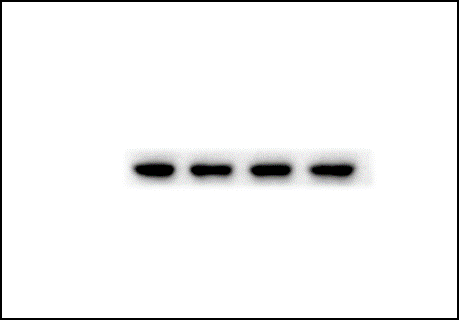

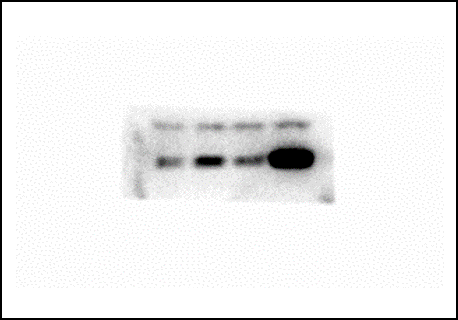

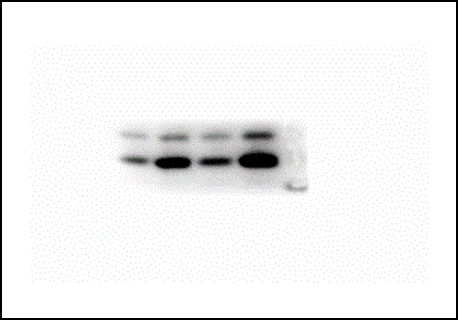

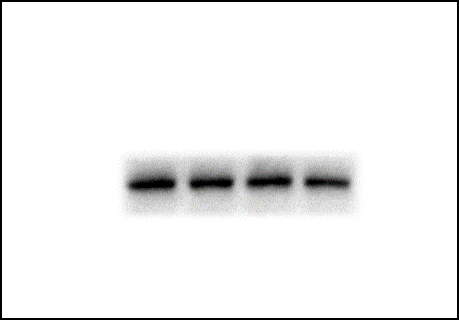

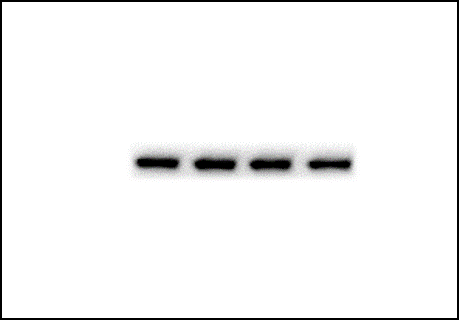

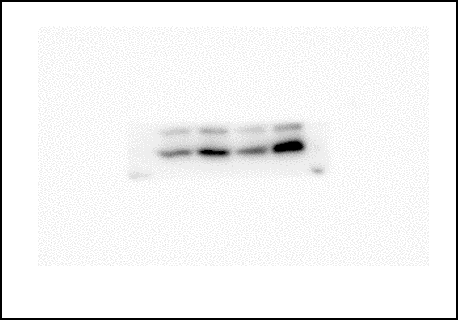


LC3

β-Actin

LC3

β-Actin

LC3

β-Actin

4. Original images of Figure 6A and S4E.


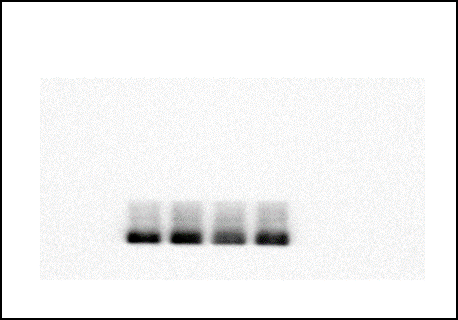

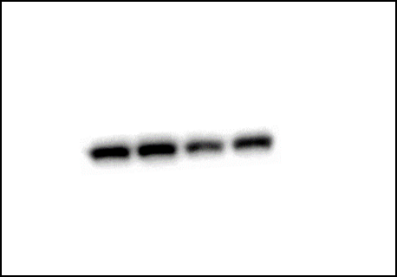

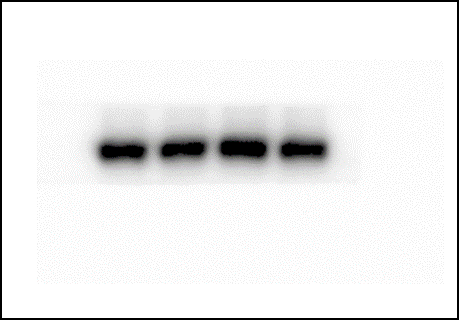

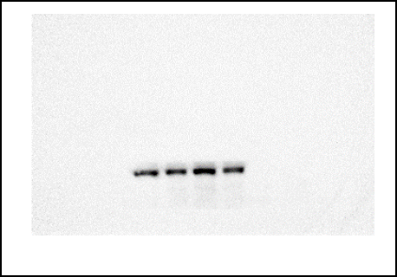

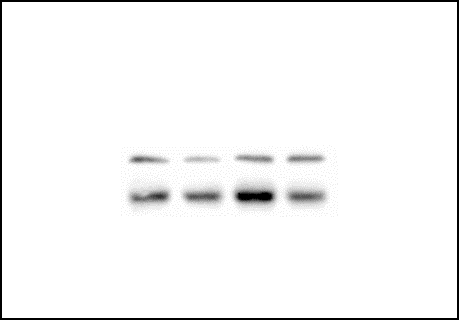

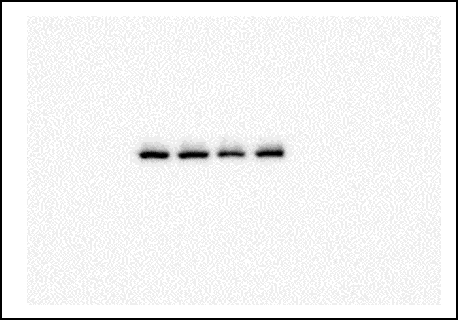


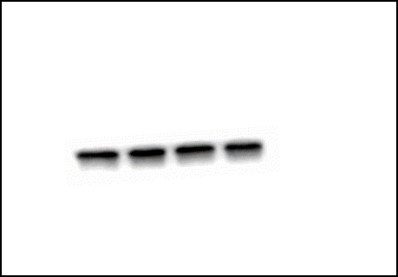

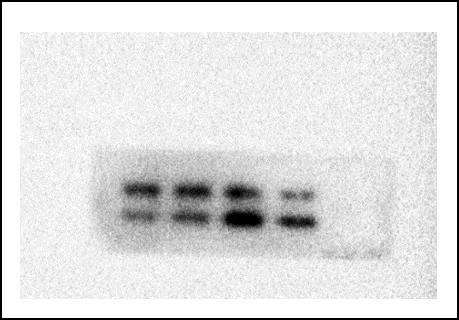

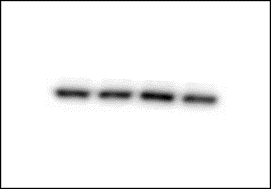

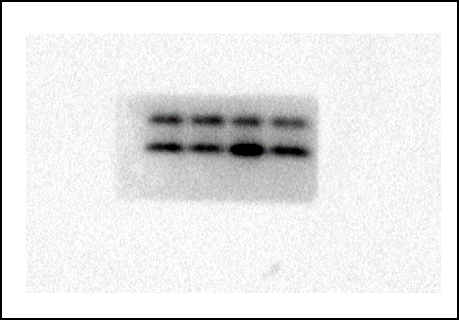

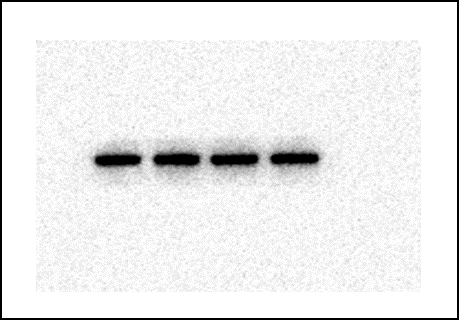

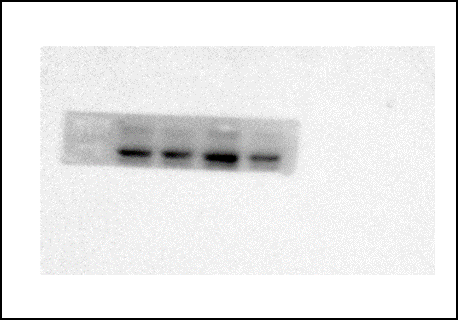


p62

Beclin-1

β-Actin

LC3

p62

Beclin-1

β-Actin

LC3

p62

Beclin-1

β-Actin

LC3

5. Original images of Figure 6E and S4G.

HGC27

MGC803

AGS

PARP

Pro caspase 9


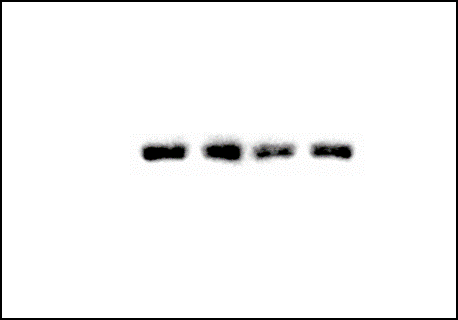

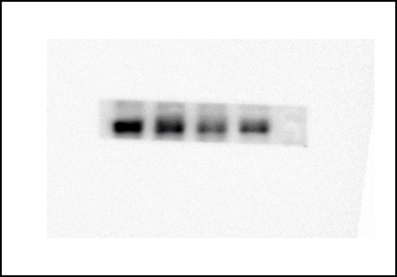

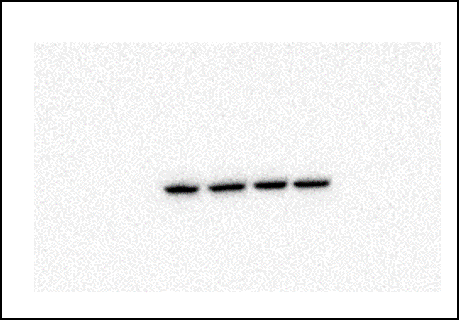

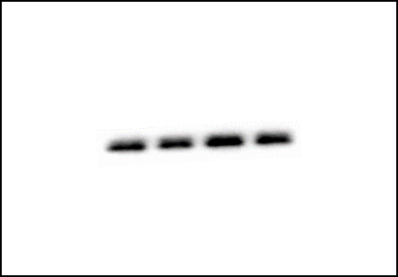

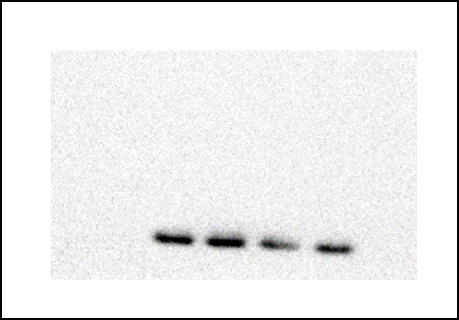

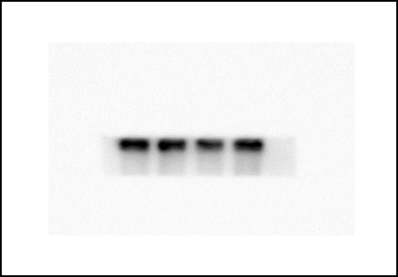

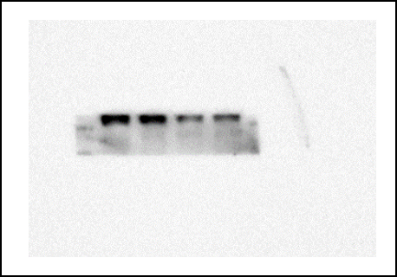

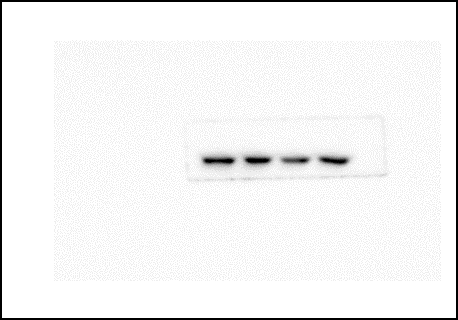
.

HGC27

MGC803

AGS

PARP

Pro caspase 9

PARP

Pro caspase 9

PARP

Pro caspase 9


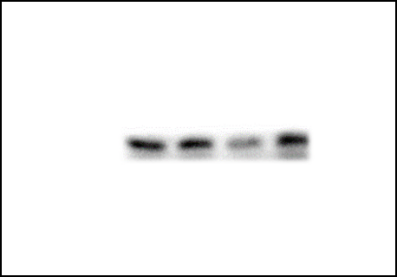

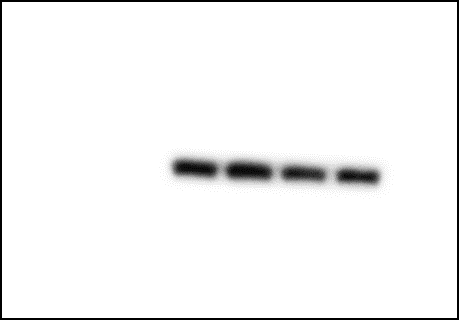

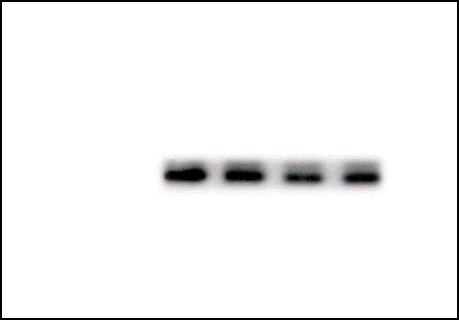

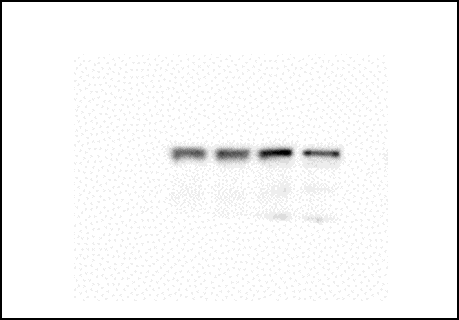

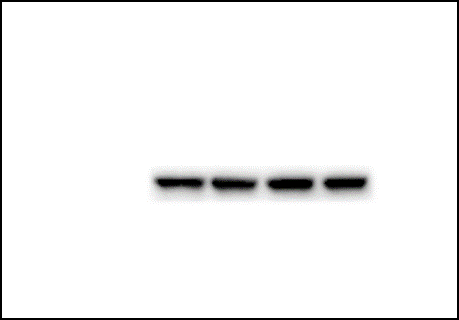

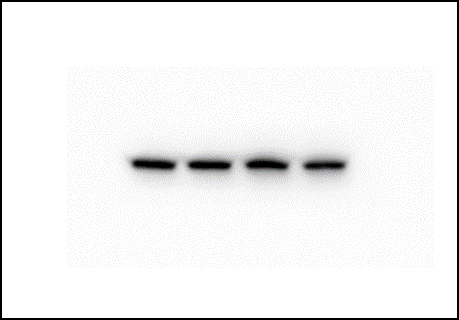

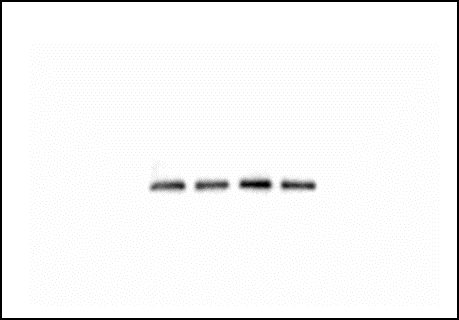

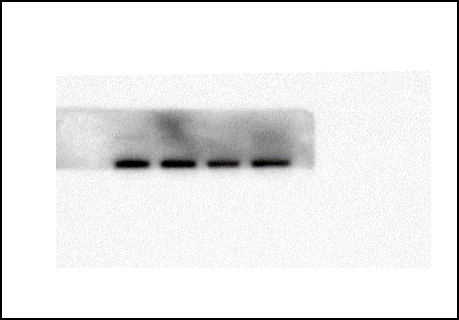

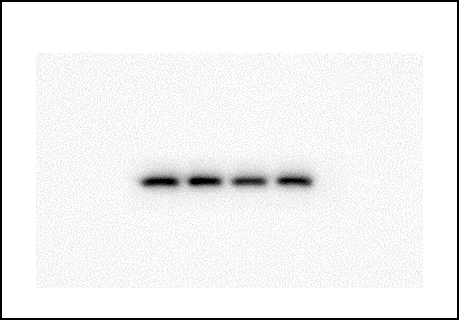

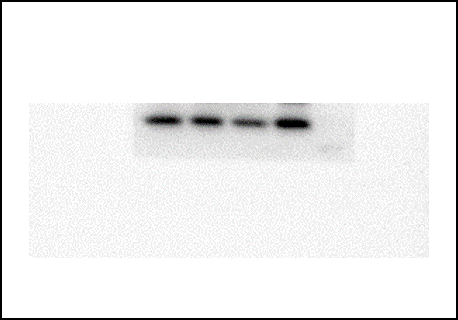


Pro caspase 3

Bcl-2

β-Actin

Bax

Pro caspase 3

Bcl-2

β-Actin

Bax

Pro caspase 3

Bcl-2

β-Actin

Bax


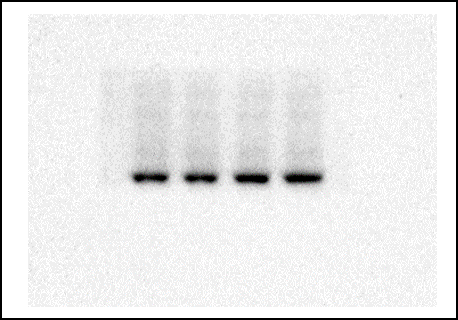

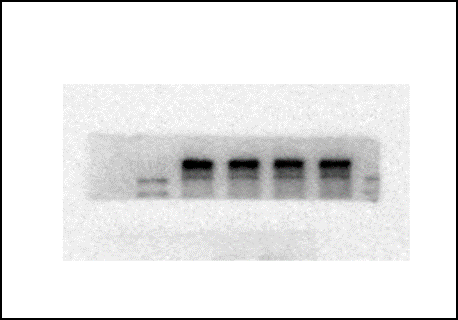

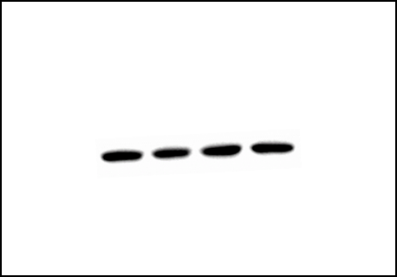

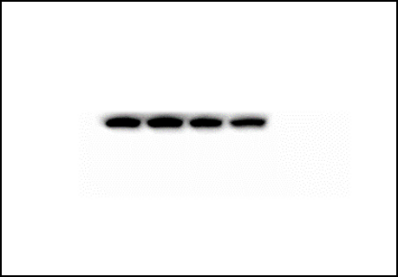

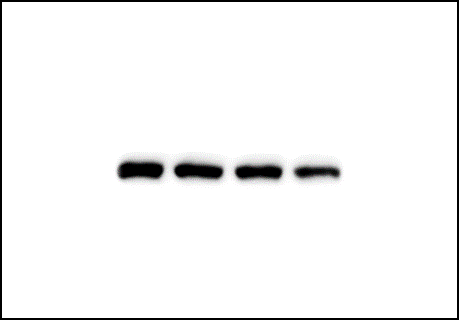

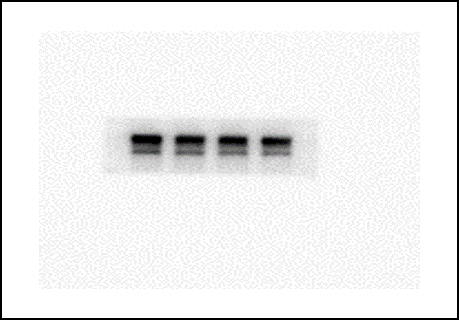

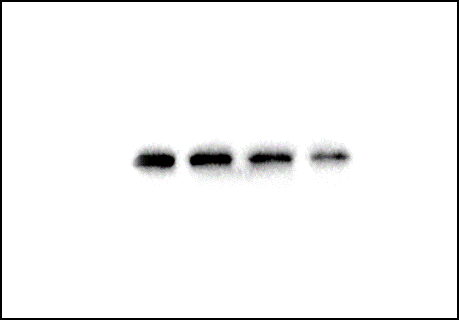

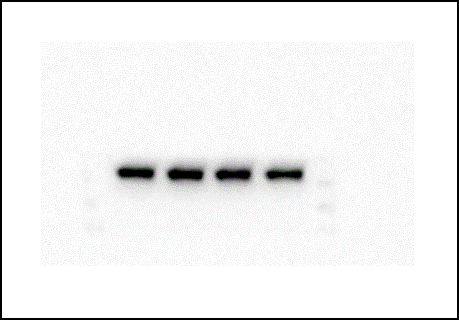

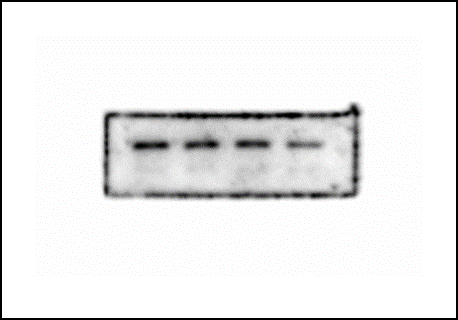

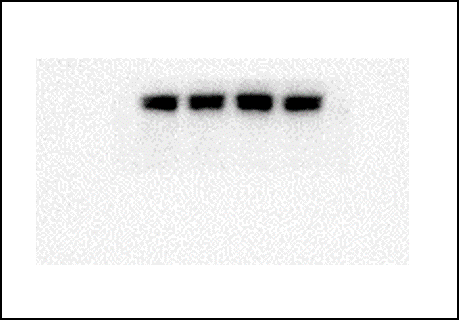

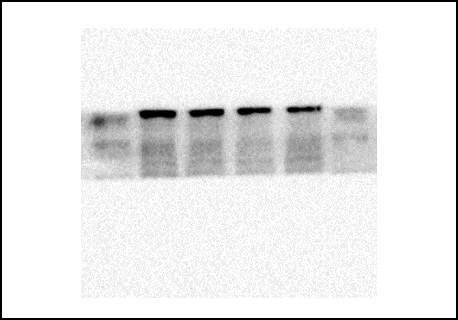

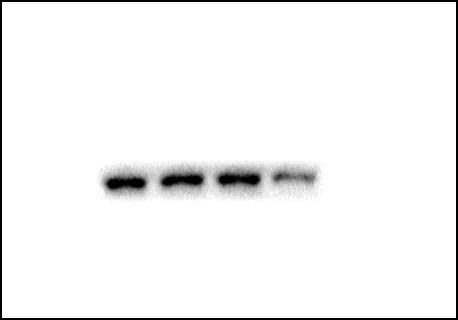

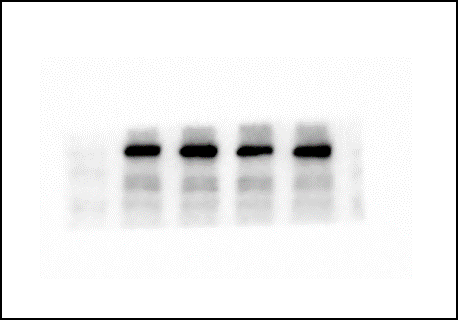

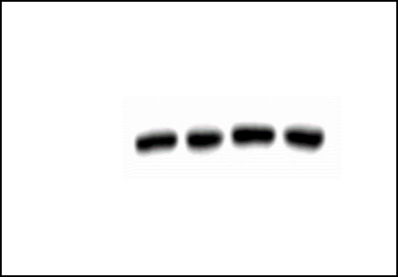

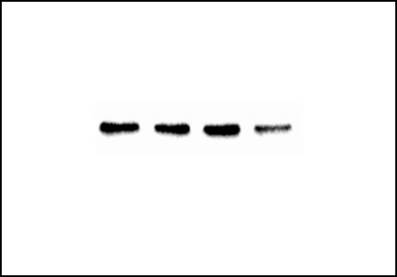

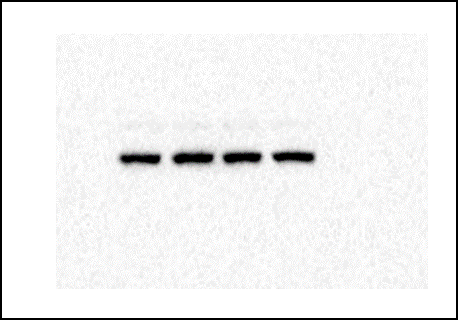

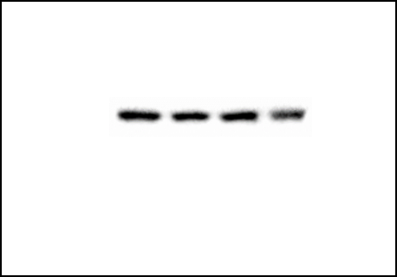

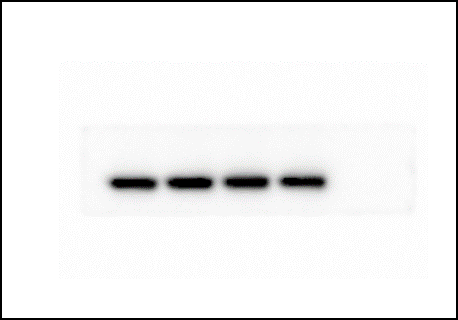

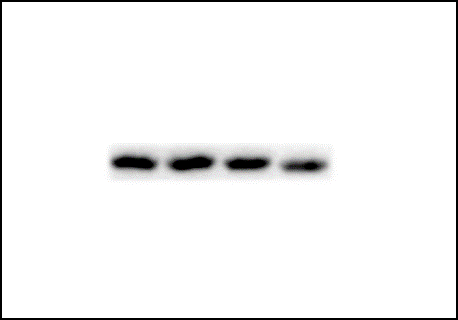

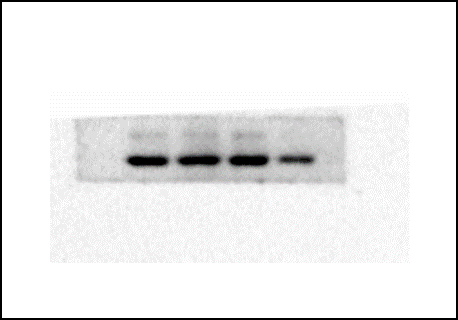
6. Original images of Figure 7B and S5A.

p-AKT

mTOR

p-PI3K

PI3K

p-AKT

mTOR

β-Actin

p-PI3K

PI3K

p-AKT

mTOR

β-Actin

p-PI3K

PI3K

AGS

MGC803

HGC27


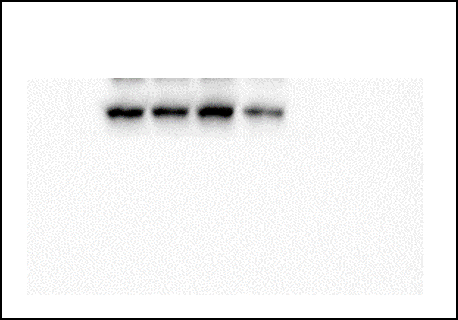


β-Actin

p-mTOR

p-mTOR

p-mTOR

AKT

AKT

AKT

7. Original images of Figure 7D and S5C.

HGC27


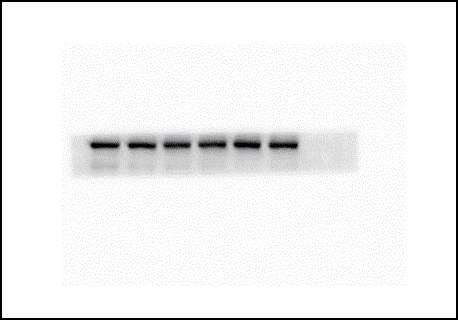

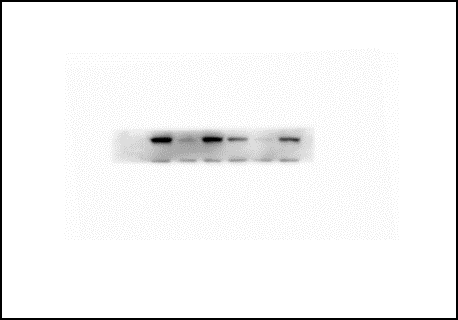

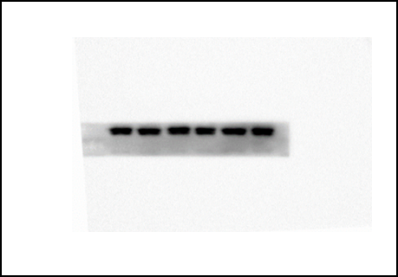

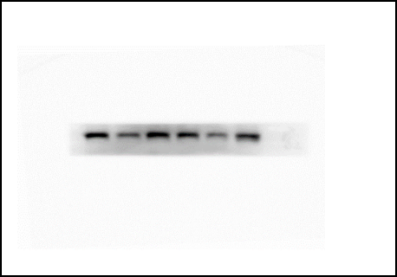

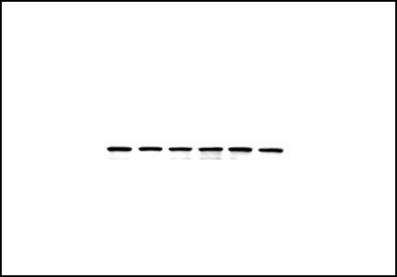

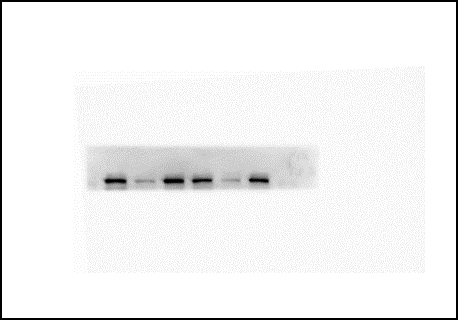

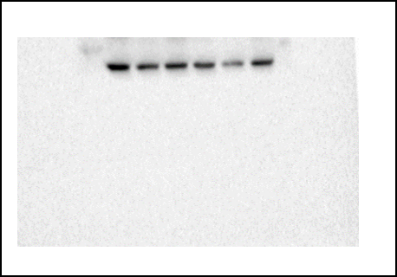

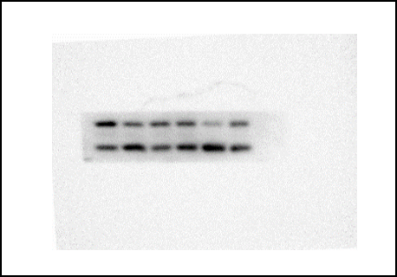


AKT

p-PI3K

PI3K

p-mTOR

mTOR

p-AKT

p62

LC3

β-Actin

MGC803

p-mTOR

mTOR

p-AKT

AKT

p-PI3K

PI3K

p62

LC3

β-Actin

AGS

β-Actin

LC3

p62

PI3K

p-PI3K

AKT

p-AKT

mTOR

p-mTOR

8. Original images of Figure 7F and S5C.

HGC27

β-Actin

LC3

p62

PI3K

p-PI3K

AKT

p-AKT

mTOR

p-mTOR

MGC803

β-Actin

LC3

p62

PI3K

p-PI3K

AKT

p-AKT

mTOR

p-mTOR

AGS

β-Actin

LC3

p62

PI3K

p-PI3K

AKT

p-AKT

mTOR

p-mTOR
